# Cardiovascular magnetic resonance physics for clinicians: part II

**DOI:** 10.1186/1532-429X-14-66

**Published:** 2012-09-20

**Authors:** John D Biglands, Aleksandra Radjenovic, John P Ridgway

**Affiliations:** 1 Division of Medical Physics, University of Leeds, Leeds, UK; 2 NIHR-Leeds Musculoskeletal Biomedical Research Unit and Leeds Institute of Molecular Medicine, University of Leeds, Leeds, UK; 3 Department of Medical Physics and Engineering, Leeds Teaching Hospitals NHS Trust, 1st Floor, Bexley Wing, St James's University Hospital, Leeds, LS9 7TF, UK; 4 Multidisciplinary Cardiovascular Research Centre, University of Leeds, Leeds, UK

**Keywords:** Cardiovascular magnetic resonance, MR physics, MR contrast agents, Fat suppression, Tagging, Myocardial perfusion imaging, Late gadolinium enhancement, MR angiography, Velocity mapping, Myocardial edema, Myocardial ischemia, Coronary heart disease, Myocardial infarction

## Abstract

This is the second of two reviews that is intended to cover the essential aspects of cardiovascular magnetic resonance (CMR) physics in a way that is understandable and relevant to clinicians using CMR in their daily practice. Starting with the basic pulse sequences and contrast mechanisms described in part I, it briefly discusses further approaches to accelerate image acquisition. It then continues by showing in detail how the contrast behaviour of black blood fast spin echo and bright blood cine gradient echo techniques can be modified by adding rf preparation pulses to derive a number of more specialised pulse sequences. The simplest examples described include T2-weighted oedema imaging, fat suppression and myocardial tagging cine pulse sequences. Two further important derivatives of the gradient echo pulse sequence, obtained by adding preparation pulses, are used in combination with the administration of a gadolinium-based contrast agent for myocardial perfusion imaging and the assessment of myocardial tissue viability using a late gadolinium enhancement (LGE) technique. These two imaging techniques are discussed in more detail, outlining the basic principles of each pulse sequence, the practical steps required to achieve the best results in a clinical setting and, in the case of perfusion, explaining some of the factors that influence current approaches to perfusion image analysis. The key principles of contrast-enhanced magnetic resonance angiography (CE-MRA) are also explained in detail, especially focusing on timing of the acquisition following contrast agent bolus administration, and current approaches to achieving time resolved MRA. Alternative MRA techniques that do not require the use of an endogenous contrast agent are summarised, and the specialised pulse sequence used to image the coronary arteries, using respiratory navigator gating, is described in detail. The article concludes by explaining the principle behind phase contrast imaging techniques which create images that represent the phase of the MR signal rather than the magnitude. It is shown how this principle can be used to generate velocity maps by designing gradient waveforms that give rise to a relative phase change that is proportional to velocity. Choice of velocity encoding range and key pitfalls in the use of this technique are discussed.

## Review

This review is the second part of two that aim to cover the basic physical principles underlying the most commonly used cardiovascular magnetic resonance CMR) techniques. In part I, the basic principles of MR signal generation and image formation were reviewed, together with the principles of cardiac synchronisation and fast (or turbo) imaging pulse sequences and how these can be combined to achieve imaging of the heart within a single patient breath-hold [1]. Part I is concluded by describing the two most commonly used cardiac MR imaging techniques; anatomical imaging using a double inversion, black-blood spin echo pulse sequence and bright blood functional cine imaging using either spoiled gradient echo or balanced steady state free precession (bSSFP) imaging pulse sequences.

Part II of this review aims to cover the remaining imaging techniques that are commonly used in cardiac MR imaging. There are many excellent texts that provide further in-depth coverage of the techniques discussed here [2-9]. Each technique can be considered as being based upon either the spin echo or gradient echo pulse sequences already described in part I, but with certain modifications applied to their contrast behaviour or mode of acquisition. Three main approaches to the modification of contrast behaviour are discussed. Firstly, it is shown how radiofrequency (rf) preparation pulses can be added to existing pulse sequences to alter their contrast behaviour: An inversion pulse added to a black-blood FSE/TSE pulse sequence enhances T1 and T2 weighted contrast for imaging of myocardial oedema [10]. A fat suppression pulse added to an existing pulse sequence selectively suppresses the MR signal contribution from lipid based tissues [11], thus improving the delineation of non-lipid based structures. More complex preparation pulses may also be added to the cine gradient echo pulse sequence to apply a line or grid pattern to achieve myocardial tissue tagging that allows visualisation of intra-myocardial motion [12,13].

The second method of modifying the MR contrast behaviour is through the intravenous administration of an endogenous contrast agent, based on the paramagnetic Gadolinium (Gd) ion [14]. This contrast agent is used in two of the key CMR applications for the imaging of ischaemic heart disease; myocardial perfusion imaging [15,16] and late gadolinium enhancement (LGE) imaging [17,18]. Both of these techniques combine the use of preparation pulses and the administration of a Gd-based contrast agent to achieve T1 contrast weighting. The use of contrast agent also provides the basis of contrast enhanced MR angiography (CE-MRA) which is now in widespread use to image most major vessels outside the heart [19]. Magnetic resonance angiography techniques that do not make use of Gd-based contrast agent are also summarised. Imaging of the coronary arteries is most commonly performed without the use of contrast agent. The most common approach, performed with the patient free-breathing and using navigator echoes for gating of the respiratory cycle [20,21], is described in detail.

The third contrast mechanism that is discussed in this review differs somewhat from the first two. Whereas for all of the techniques discussed so far, the MR image pixel intensity depends on the magnitude of the MR signal intensity, for phase contrast techniques the image pixel intensity is related to the phase of the MR signal. In this section it will be shown how the imaging gradients can be used to encode the velocity of blood flowing along a particular gradient direction to generate a relative phase change that is proportional to velocity [22-24]. This provides a quantitative measure of blood velocity and blood flow and has particular application in valvular and congenital heart disease.

All of the above techniques make use of the segmented k-space fast imaging techniques (either turbo/fast spin echo or turbo/fast gradient echo) described in part I to ensure that image acquisition can be performed within an acceptable breath-hold period, or in the case of perfusion imaging, within a single heart beat. A number of other acceleration techniques are used to further reduce acquisition times to provide shorter breath-hold periods or to improve temporal and spatial resolution. Although a detailed description of these techniques is beyond the scope of this review, they are briefly summarised in the following section.

### More acceleration techniques

**Figure 1:**
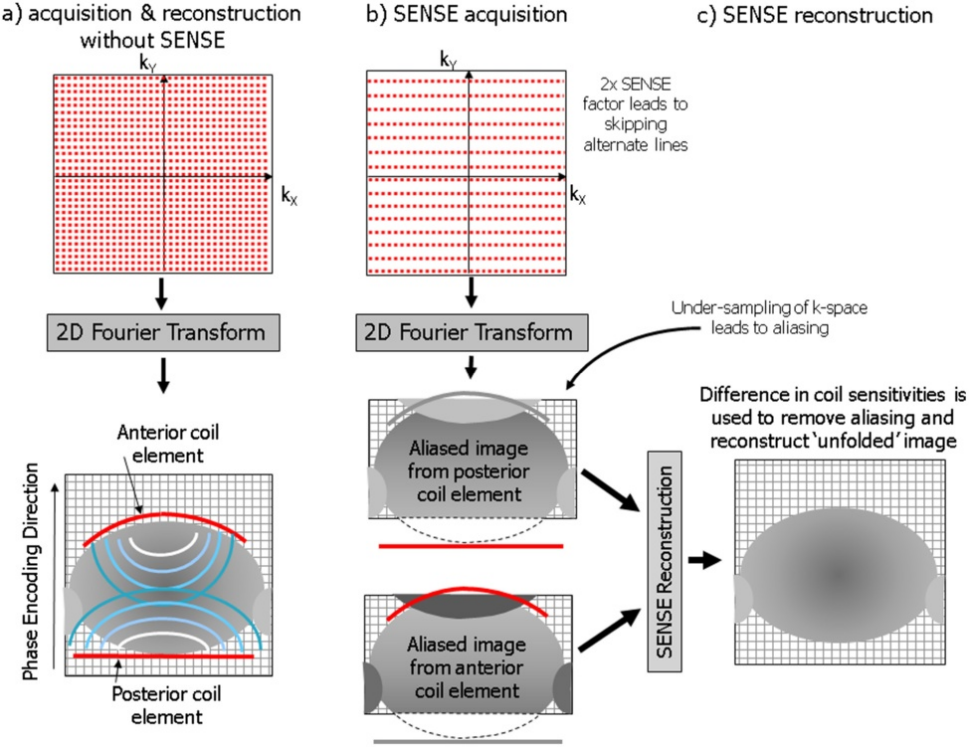
**Parallel imaging.** Parallel imaging uses the spatial distribution of coil array elements and their characteristic sensitivity maps to provide spatial information. This allows under-sampling of k-space (skipping of phase encoding steps) during the acquisition, shortening the image acquisition time. (**a**) shows a full k-space acquisition without parallel imaging. Image signal intensities from anterior and posterior coil elements are combined in phased-array mode to provide uniform signal intensity across the field of view. (**b**) Parallel imaging using the SENSE method with a reduction factor of 2 (2x SENSE) leads to skipping of alternate lines of k-space. Without the SENSE reconstruction this would lead to an effective reduction of the field of view and image aliasing or ‘foldover’. As an intermediate reconstruction step, separate aliased images are obtained for each coil element. In (**c**) the SENSE reconstruction uses the images obtained separately from the posterior and anterior coil elements, together with the coil element sensitivity maps acquired from a reference scan to remove the aliasing and reconstruct an unfolded the image with a full field of view.

The acceleration techniques described here and summarised in Table 1 all involve a reduction in the number of phase encoding steps, and therefore the acquired number of k-space lines, to achieve the reduction in acquisition time. Some of these techniques have been in long-standing widespread use, such as the reduction of image acquisition matrix size and/or field of view in the phase encoding direction [25,26] and the acquisition of data for only just over half of k-space, exploiting the symmetry property of k-space [25,27]. More complex k-space under-sampling techniques, in which additional lines of k-space can be omitted where there is redundancy of information, have come into use. The most established of these techniques, parallel imaging [28-30], was made possible with the adoption of multi-element rf receiver coil arrays. Parallel imaging makes use of the geometric distribution of the array coil elements to restore information that is lost by acquiring a reduced number of phase encoding steps (known as k-space under-sampling) (Figure 1). The number of acquired phase encoding steps is reduced by a certain factor, known as the reduction factor, R. MR signal data covering the same extent of k-space is acquired, preserving the spatial resolution, but fewer lines of k-space are acquired, spaced further apart (Figure 1b). This would normally result in aliasing of information in the phase encoding direction with signal wrapping round from one side of the image to the opposite side. The distribution of at least two elements of an rf coil array, along the phase encoding direction provides information about the true location of the signal. The reconstruction process requires knowledge of the coil sensitivity, a measure of how the detected signal intensity varies with distance from each coil element. Full image reconstruction is achieved by using the signal from each coil element, together with the coil sensitivity information to produce an image without aliasing (Figure 1c). This reconstruction step can either be performed in the image space (SENSE, ASSET) or in k-space (SMASH, GRAPPA, ARC). A knowledge of the coil sensitivity map or signal intensity distribution for each patient, coil array element and image slice geometry is essential for this technique to work. Sensitivity maps are formed from the central lines of k-space and can either be acquired as a separate reference scan (as with SENSE or ASSET), or concurrently as part of the acquisition (mSENSE, GRAPPA, ARC). 

**Table 1:** Acceleration Techniques

	**Reduced matrix in the phase encoding direction**	**Reduced field of view in the phase encoding direction**	**Half -Fourier acquisition**	**Parallel Imaging**		**Temporal under-sampling**
				**Image-based reconstruction**	**k-space-based reconstruction**	
Common names	Scan %	Rectangular FOV (RFOV)	Half Fourier	SENSE	SMASH	View-sharing
				mSENSE*	GRAPPA*	Keyhole
				ASSET	ARC*	UNFOLD
				SPEEDER		TSENSE
						Kt-BLAST
	Phase resolution		Partial Fourier			
			Half Scan			
			0.5 NEX			
		Phase FOV				
		Asymmetric FOV				

	Zero Padding					
Reference Number	[25,26]	[26]	[25,27]	[25,28,29]	[25,28,30]	[25,28,31,32]

*Self calibrating (auto-calibrating) techniques – reference data acquired as part of acquisition.

Summary of acceleration techniques where the data acquisition time is shortened by reducing the number of phase encoding steps.

Parallel imaging techniques are mainly used to reduce imaging time, especially to shorten breath-hold periods, but may also be used to improve either temporal or spatial resolution for the same imaging time. There are however drawbacks of parallel imaging: The image signal-to-noise ratio is reduced according to the square root of the reduction factor. For a reduction factor of 2, this results in a reduction of the signal-to-noise ratio by approximately 30 %, and so parallel imaging should only be applied when the image signal-to-noise ratio is sufficient to accommodate such a reduction. Furthermore, if the field of view in the phase encoding direction is set too small, residual aliasing or foldover artefacts will appear at the centre of the image which cannot be removed by the parallel imaging reconstruction, so careful selection of the field of view and image orientation is needed. The phase encoding direction in which the parallel imaging reduction factor is applied must also always be chosen along a direction where there is a favourable distribution of array coil elements. Otherwise the reconstruction will fail leading to high noise levels at the centre of the image.

More recently k-space under-sampling techniques that make use of information that is repeated over time have been developed for cine and dynamic imaging [31,32] and are being translated into products.

### Adding preparation pulses to modify contrast

#### Preparation pulses – general principles

**Figure 2:**
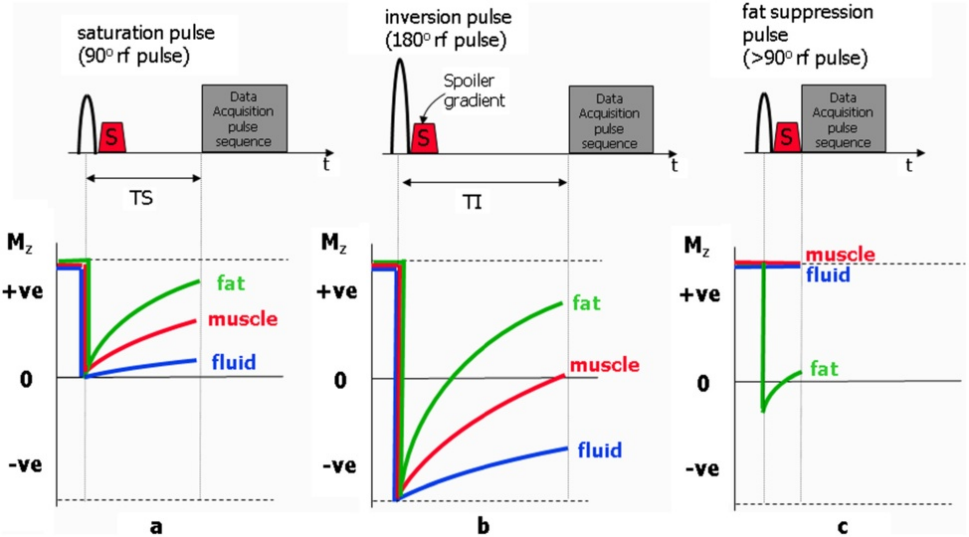
**The three most commonly used magnetisation preparation pulses, showing their relation to the image data acquisition pulse sequence.** Beneath each pulse sequence, curves show the behaviour of the z-magnetisation for three different tissues (fat, muscle and fluid). Spoiler gradients, S are applied to suppress any unwanted transverse magnetisation produced by the preparation pulses. In (**a**) following the 90° saturation pulse the z-magnetisation for all tissues is reduced to zero and recovers according to their T1 relaxation rate. When the imaging pulse sequence is applied after a delay TS the resultant contrast is T1-weighted with the shortest T1 (fat) yielding the highest signal intensity. In (**b**) following the 180° inversion pulse the z-magnetisation for all tissues is inverted, and then recovers from a negative value. When the imaging pulse sequence is applied after a delay TI, the tissue for which its z-magnetisation is passing through zero yields no signal, effectively suppressing the signal contribution from that tissue. In this example, the signal from muscle is suppressed. In (**c**) The frequency-selective fat suppression pulse is applied only at the resonant (Larmor) frequency of fat so that only the z-magnetisation of fat-based tissue is reduced. In this case the imaging pulse sequence is applied with no delay. The z-magnetisation of other tissues (muscle and fluid) are unaffected by the fat suppression pulse and will yield normal signal levels.

**Figure 3:**
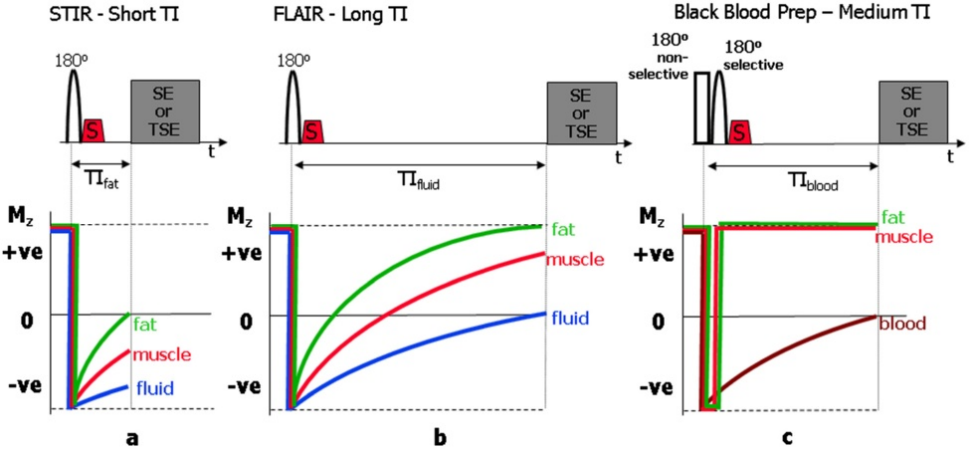
**STIR, FLAIR and Black-Blood Preparation schemes.** Pulse sequence timing and z-magnetisation curves for the three common special cases of the inversion recovery preparation scheme. In (**a**) the short TI inversion recovery (STIR) pulse sequence has the TI chosen to correspond to the null point for fat tissue (TI_fat_) resulting in the suppression of signal from fat. Note that for a modulus reconstruction, the sign of the magnetisation is ignored and the displayed signal intensity from the other tissues increases with increasing T1 value, with fluid yielding the highest signal intensity. In (**b**) the fluid attenuated inversion recovery (FLAIR) pulse sequence has the TI chosen to be at the null point for fluid to suppress the signal from fluid (TI_fluid_). In (**c**) the first inversion pulse of the black-blood preparation scheme has its TI chosen to correspond to the null point for blood (TI_blood_). This pulse is non-slice-selective and so causes the inversion of magnetisation for all tissues. The second slice-selective inversion pulse is needed to undo the inversion applied to the tissues within the imaged slice (myocardial muscle, fat), otherwise their signals would also be suppressed. The combined effect of the two inversion pulses is therefore to invert the tissues including blood outside the imaged slice. The subsequent wash-in of inverted blood into the image slice during the TI period results in suppression of signal from the blood pool.

Preparation pulses are rf pulses that are combined with either spin echo or gradient echo pulse sequences to modify their contrast behaviour. In part I of this review it was shown how the MR signal produced by a spin echo or gradient echo pulse sequence depends upon the value of the net magnetisation immediately prior to each repetition of the pulse sequence. This depends on factors such as the flip angle of the rf excitation pulse, the time between each repetition (the repetition time, TR) and the rate at which the net magnetisation returns to its starting value at equilibrium (determined by T1 relaxation with a characteristic time constant, T1, for a particular tissue). Preparation pulses are applied in order to further prepare the net magnetisation before the standard pulse sequence is applied, a process known as magnetisation preparation. The most common preparation pulses are saturation pulses, inversion pulses and frequency selective fat-suppression pulses (Figure 2). Saturation and inversion pulses are typically followed by a prescribed time delay to allow recovery of the prepared magnetisation before a spin echo or gradient echo pulse sequence is used to ‘read out’ the MR signal. These techniques are known as saturation recovery and inversion recovery respectively and the associated delay times are known as the time from saturation, TS, and the time from inversion, TI (Figure 2a and Figure 2b). Frequency selective fat-saturation pulses are applied immediately before the readout pulse sequence to minimise recovery of the net magnetisation arising from fat-based tissues (Figure 2c). In general radiology applications, the inversion recovery technique is commonly employed to suppress signal from selected tissues. This is done by choosing the inversion time to coincide with the time at which the recovering net magnetisation of a particular tissue passes through zero, the null point (see Figure 2b). For non-cardiac applications, the two most common instances of the inversion recovery technique are Short TI Inversion Recovery (STIR) [33,34] with the TI chosen to correspond to the null point for fat tissue (Figure 3a), thus resulting in the suppression of signal from fat, and Fluid Attenuated Inversion Recovery (FLAIR) [35] with the TI chosen to be at the null point for fluid (Figure 3b), specifically, to suppress the signal from cerebrospinal fluid for applications in the brain. For cardiac applications, this same principle is used when applying the black blood preparation scheme, described in Part I [1]. This preparation scheme is used to improve the suppression of blood signal in black blood anatomical imaging with fast or turbo spin echo pulse sequences. In this case, the TI is chosen to correspond to the null point for blood (Figure 3c), however two 180° preparation pulses are needed for if only a single inversion is used, the signal from the myocardium within the imaging slice would also be partially suppressed. The combined application of non-selective and selective pulses causes only the blood outside the slice to be inverted, leaving the myocardium to produce a signal that is unmodified by the preparation scheme. 

### STIR, Triple IR and oedema imaging

**Figure 4:**
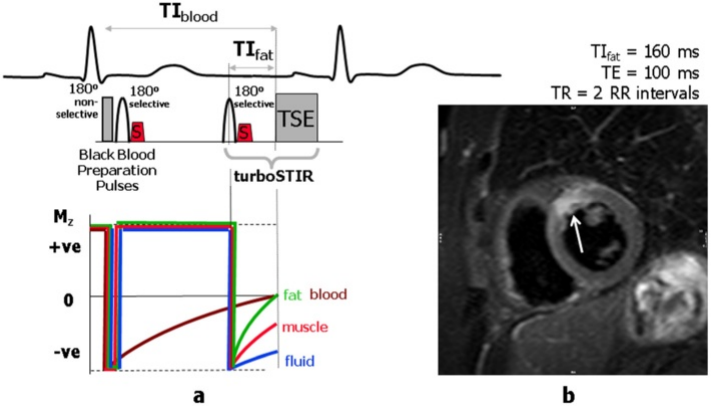
**Triple IR Preparation scheme for Oedema imaging.** Triple inversion recovery TSE (Black-Blood turboSTIR) pulse sequence commonly used for oedema imaging. In (**a**) the pulse sequence timing is shown together with z-magnetisation curves. The preparation scheme consists of the two 180° pulses for the black blood preparation, followed by a third slice-selective 180° pulse to provide the STIR contrast. The sequence has two inversion times, TI_blood_ and TI_fat_. TI_fat_ has the same value as that used for fat suppression as in the STIR sequence (approx 160 milliseconds). Calculation of the value for TI_blood_ depends on the heart rate, and the effective TR (number of heart beats per repetition). The z-magnetisation curve shown for blood assumes it washes into the slice after the third 180° pulse has been applied. The image in (**b**) is acquired in the short axis plane using the black-blood turbo STIR technique. The intrinsic fluid weighting of the STIR technique has been further enhanced by selecting a long TE value (100 ms) to introduce T2-weighting (the technique is sometimes referred to as T2-weighted STIR). Myocardial oedema can be seen as an area of increased signal (arrow). (Image courtesy of Darach O h-lci and Daniel Messroghli, Deutsches Herzzentrum, Berlin).

In general radiology applications the STIR technique is useful not only because it suppresses the signal from fat. The inversion pulse applied with a short TI value reduces the signal intensity from tissues with a shorter T1 so that tissues with higher T1 relaxation times exhibit a relatively higher signal magnitude. Because this contrast behaviour (increasing signal with increasing T1) is different from that of standard T1-weighted spin echo techniques, it can also be combined with T2 weighting by increasing the echo time, TE, to further enhance the contrast where there is both an increased T1 and T2, for example, in tissue oedema. The STIR technique requires a long repetition time (similar to that required to achieve T2 weighting for spin echo sequences) so although STIR imaging can be applied using a standard spin echo pulse sequence for data acquisition, this results in very long acquisition times. It is therefore more common to combine the STIR technique with fast or turbo spin echo pulse sequences in order to reduce the acquisition time to acceptable limits and this technique is often referred to as turboSTIR. For cardiac applications, the TR is defined by triggering from every second or third heart beat, and the black blood preparation scheme is added to suppress the signal from blood (Figure 4a). The resultant black blood turbo STIR pulse sequence has strong fluid weighting but with no signal from blood within the cardiac chambers and it is particularly useful for the assessment of myocardial oedema (Figure 4b). It consists of the two 180° pulses for the black blood preparation, followed by a third, normally slice-selective, 180° pulse to provide the STIR contrast [10]. It is therefore sometimes known as a triple inversion recovery pulse sequence. The sequence has two inversion times, TI_Blood_ and TI_fat_. TI_fat_ has the same value for fat suppression as in the STIR sequence (approx 160 milliseconds). Calculation of the value for TI_Blood_ is more complicated and depends on the heart rate and the number of heart beats between trigger pulses. Some vendors enable the operator to select the third 180° pulse as either a slice-selective or non-slice -selective pulse, and this must be taken into account in the calculation of TI_Blood_. The setting of other imaging parameter values is similar to the T2-weighted Black Blood FSE/TSE pulse sequence described in Part 1 [1]. 

### Frequency selective fat suppression

**Figure 5:**
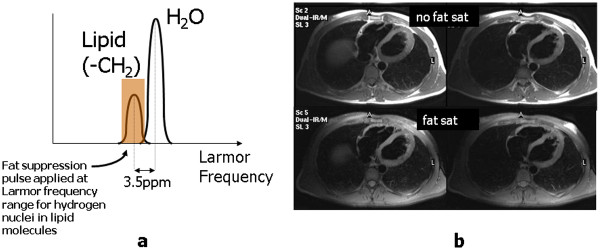
**Frequency selective fat suppression.** MR signal contributions from lipid and water molecules are plotted in (**a**) as a function of Larmor frequency. Both the lipid and water populations exhibit a small range of Larmor frequencies due to the existence of spin-spin interactions, however the central frequency of the lipid population is shifted to a lower frequency than that of water due to the increased shielding effect of the electron cloud surrounding the larger lipid molecule. To achieve fat suppression, a frequency-selective rf pulse (Chemical Shift Selective or CHESS pulse) is applied at the Larmor frequency of the lipid population, causing saturation of the lipid magnetisation. The image data acquisition pulse sequence is then applied immediately after the fat suppression pulse (Figure 2c). In (**b**) two axial slices acquired using a black blood TSE pulse sequence are shown without (top row) and with (bottom row) fat suppression.

An alternative method of achieving suppression of the signal from fat-based tissue is to exploit the difference in Larmor frequency between the hydrogen nuclei in lipid molecules and water molecules [33,36]. This difference, known as the chemical shift, is equal to 3.5 parts per million of their Larmor frequency (Figure 5a). At 1.5 T this is a difference of approximately 220 Hz. The signal from the lipid molecules is suppressed by applying a 90° rf pulse at the Larmor frequency of fat (Figure 5a). As no magnetic field gradient is applied, this pulse will saturate (reduce to zero) the z-magnetisation of fat within the entire imaging volume, provided that the Larmor frequency is constant (i.e. the magnetic field is uniform throughout the imaging volume). The transverse magnetisation produced by the rf pulse is de-phased using a spoiler gradient and the pulse sequence used for image data acquisition is then applied immediately afterwards (Figure 2c). As the fat magnetisation is already saturated by the fat suppression pulse, it does not contribute any signal and appears on the image as a signal void. This frequency-selective method of fat suppression is also known as chemical shift selective (CHESS) imaging [36]. For fat suppression to be achieved uniformly across the whole image, there is a particular requirement for the magnetic field to be uniform to a much higher specification than is normally required for imaging (although Balanced SSFP imaging also requires a similarly high uniformity). In addition to intrinsic variation in the magnetic field, there are also patient-induced variations that require patient-specific adjustment of the magnet field uniformity. This adjustment, know as dynamic shimming, is performed as part of the pre-scan adjustments by adding small gradient magnetic fields applied using the gradient coils, and on some MR systems an additional set of dedicated shim coils. Frequency selective fat suppression has the advantage that it can be applied to any pulse sequence (gradient or spin echo, T1- or T2-weighted) without altering the intrinsic contrast behaviour, other than the suppression of fat signal (Figure 5b). As the success of this technique relies on a uniform magnetic field, it does not work well in patients with metallic implants located close to the region of interest. It is used in clinical applications where it is desirable to suppress the high signal intensity from fat-based structures that would otherwise mask adjacent structures or areas of increased signal intensity. Frequency selective fat suppression is often combined with T2-weighted black blood turbo spin echo as an alternative to T2-weighted black blood turboSTIR for imaging of oedema. 

### Cine myocardial tagging with spatial modulation of magnetisation (SPAMM)

**Figure 6:**
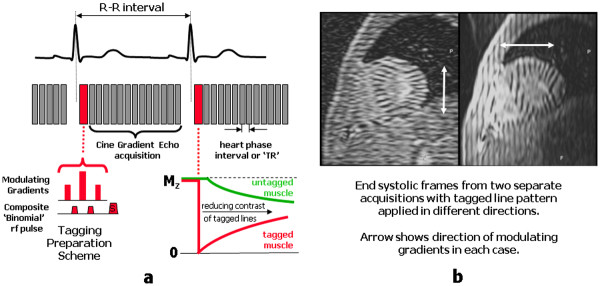
**(a) A schematic diagram of a cine tagging pulse sequence.** In this example a composite binomial rf pulse is used consisting of three rf pulses with amplitudes in the ratio of 1:2:1. Two modulating gradients are applied in the spaces between the rf pulses to de-phase (modulate) the transverse magnetisation between each rf pulse. The net effect is to cause a variation (or modulation) of the z-magnetisation, creating a series of parallel lines of tissue with a magnetisation that varies alternately between its equilibrium value (untagged) and zero (tagged). A spoiler gradient, S, is applied to destroy transverse magnetisation generated by the tagging pulses. T1 relaxation causes the magnetisation of the tagged lines to recover towards equilibrium, while at the same time the magnetisation of the untagged tissue becomes partially saturated by the rf pulses applied as part of the cine imaging sequence. This causes the contrast between the tagged and untagged lines to reduce as the cardiac cycle progresses. The two short-axis images in (**b**) are acquired from separate tagged cine acquisitions with tagging applied in two different directions. The arrows indicate the direction of the modulating gradient in each case. Both image examples correspond to a cardiac phase at around end-systole. For stationary tissue, such as in the chest wall, the tagged pattern has remained fixed and is seen as a series of parallel lines. Within the left ventricle, the line pattern has deformed as it follows the motion of the myocardial muscle.

**Figure 7:**
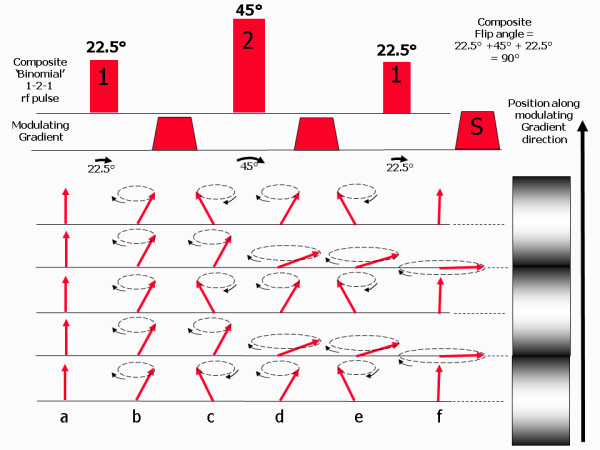
**This diagram shows how a SPAMM pulse can produce a magnetisation pattern consisting of a series of parallel lines that are alternately fully magnetised and fully saturated, appearing as bright and dark lines (shown on the right).** In this example, the composite rf pulse is a 1-2-1 binomial pulse, consisting of three rf pulses with relative amplitudes in the ratio of 1:2:1. As the effective flip angle of the composite pulse is set to be 90°, the flip angles of the three individual rf pulses is therefore 22.5°, 45° and 22.5° respectively. Starting at equilibrium (**a**), the first rf pulse causes all spins to flip through 22.5° (**b**). The first modulating gradient is then applied causing the spins to move out of phase along the gradient until there is a 180° phase shift between points that correspond to the desired spacing between adjacent bright and dark lines. The second 45° rf pulse is then applied, causing the magnetisation that is 180° out of phase to be flipped from −22.5° to +22.5°, while magnetisation that is in phase is flipped from +22.5° to 67.5° (**d**). The second modulating gradient introduces a further 180° phase shift between adjacent bright and dark tag lines (**e**). The third 22.5° rf pulse is then applied. This causes the magnetisation that is 180° out of phase to be flipped from −22.5° to 0 (aligned along the z-axis), while magnetisation that is in phase is flipped from 67.5° to 90° to become saturated (**f**).

Imaging of heart wall motion is routinely performed using cine gradient echo pulse sequences. In addition, it is possible to assess intra-myocardial motion by ‘tagging’ the myocardium at end diastole with a line or grid pattern, which then deforms as the heart wall contracts [37]. Myocardial tagging is achieved using a specialised preparation scheme consisting of series of non-selective rf preparation pulses, (together known as a composite or binomial rf pulse) combined with a series of gradient pulses, known as modulating gradients (Figure 6a). The effective flip angle of the composite rf pulse is around 90°. The modulating gradients are applied in between the rf pulses and along a direction that is parallel to the image slice. The tagging preparation scheme is applied immediately after the R-wave (at end diastole) and superimposes a pattern across the image slice consisting of lines of tissue where the magnetisation is alternately saturated or at equilibrium (Figure 7). This is commonly referred to as tagging or spatial modulation of magnetisation (SPAMM). The simplest tagging pulse consists of two rf pulses either side of a single modulating gradient [37]. The addition of more rf pulses and modulating gradients makes the line pattern sharper [38]. Immediately after the tagging preparation scheme, a cine image data acquisition is then performed using a fast cine gradient echo pulse sequence to readout the signal at multiple time points throughout the cardiac cycle. 

On the first image of the cine series (immediately after the tagging pulse), the magnetisation pattern appears as a series of low-signal-intensity parallel lines across the image where the magnetisation has been saturated. As the heart contracts through systole the magnetisation pattern deforms as it follows the contraction of the myocardial muscle (Figure 6b). As the pattern is generated through saturation of the tissue magnetisation, T1-relaxation causes the magnetisation to return towards its equilibrium value. At the same time the tissue magnetisation at equilibrium becomes partially saturated by the rf pulses applied as part of the cine imaging sequence. These two effects cause the magnetisation of the tagged and untagged tissue to converge, resulting in a rapid loss of contrast for the tagged lines and fading of the tagging pattern during the cardiac cycle. The rate at which contrast is lost can be reduced by ensuring a low flip angle is used for the cine gradient echo pulse sequence, and by limiting the number of cine frames. Typically two line patterns are generated at right angles to form a grid pattern [38]. This can be done by using two tagging preparation pulses within the same acquisition (known as grid tagging), or by performing two separate acquisitions with line tagging at right angles, and subsequently combining the two data sets as a post-processing step.

SPAMM is the most well established of the methods used to perform CMR tagging and it is derivatives of this basic method that are implemented by the MR vendors for use in routine clinical practice, with visual assessment being the main method of analysis. There have been many further developments of CMR tagging techniques in the research domain, together with methods used to analyse the tagged images, and many of these are described in the recent review [13].

### Using exogenous contrast agents to modify contrast

#### MR Contrast Agents

MR contrast agents work by modifying the tissue properties that most directly affect image contrast appearances, namely the T1 and T2 relaxation times. The most commonly-used contrast agents exploit a property of the lanthanide ion gadolinium (Gd) known as paramagnetism. This property exists due to the presence of unpaired electrons in the outer shell of a metal ion, which cause it to become temporarily magnetised when in an externally applied magnetic field creating local magnetic fields over a short range. Gadolinium is particularly strongly paramagnetic as it has seven unpaired electrons in its outer shell, the most of any element. Local field interactions between the unpaired electrons of the Gd ion and the hydrogen nuclei within adjacent water molecules cause a reduction in both the T1 and T2 of the surrounding tissue. In order for this naturally toxic element to be suitable for use in human subjects, the Gd ion is bound or *chelated* to a larger electron-donating molecule or *ligand*. This renders the gadolinium safe for in-vivo use in most circumstances although gadolinium-based contrast agents are contraindicated for use in patients with impaired renal function due to their association with nephrogenic systemic fibrosis (NSF) [39-41]. The ability of a given contrast agent to influence relaxation rates is expressed in terms of its *relaxivity* which is the change in relaxation rate per unit concentration expressed in mM^-1 .^s^-1^. The higher the value of the relaxivity, the greater is the T1-reducing effect of the contrast medium. If the concentration in mM of contrast agent is *C* and the T1 relaxivity is *r*_*1*_ then the observed relaxation rate of the tissue *T*_*1 (observed)*_ can be related to its native relaxation rate *T*_*1(native)*_ as follows:

$$1/T_{1\;\left(\text{observed}\right)}=1/T_{1\;\left(\text{native}\right)}+r_{1}\cdot C$$

There is a corresponding expression for the observed T2 relaxation rate of the substance *T*_*2 (observed)*_ as follows:

$$1/T_{2\;\left(\text{observed}\right)}=1/T_{2\left(\text{native}\right)}+r_{2}\cdot C$$

**Figure 8:**
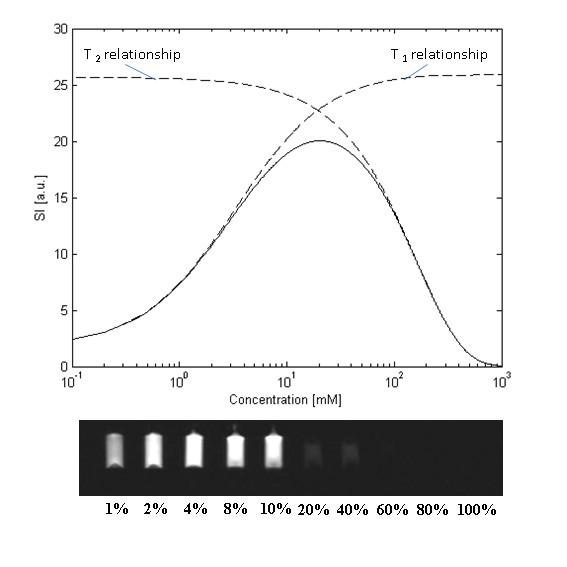
**The relationship between signal intensity and concentration.** Signal intensity values over a range of concentrations for a spoiled gradient echo pulse sequence. The two dashed curves show the separate dependencies of the signal behaviour for T1 or T2 alone. The solid line shows the combined effect of T1 or T2 on signal intensity. At low concentrations the effect of T1 shortening is dominant, while at higher concentrations the T2 shortening effect becomes the dominant factor. A series of samples imaged with increasing percentage concentrations of Gadolinium are shown underneath the plot as a visual demonstration of the effect.

Where *r*_*2*_ is the T2 relaxivity and *T*_*2(native)*_ is the native relaxation rate. Relating *T*_*1(observed)*_ to the final image signal intensity (SI) value is more complicated. SI is dependent on T1, T2, proton density, the MR imaging sequence and its parameters. Figure 8 describes a typical plot of SI versus contrast agent concentration. At low concentrations T1 shortening is the dominant effect of the contrast agent so that the SI increases with increasing concentration. However at higher concentrations the T2 shortening effect becomes dominant and SI begins to fall due to the reduction of T2 to very low values. If the purpose of the administered contrast agent is simply to enhance certain structures in the image, as in MRA investigations (see later), then the administered dose is designed to maximise the SI and seeks to produce an in-vivo Gd concentration corresponding to the peak in Figure 8. However if the images are to be used for quantitative analysis then the contrast-induced changes in SI must directly reflect changes in Gd concentration. At low concentrations this assumption holds because the relationship between SI and Gd concentration is approximately linear. At higher concentrations this relationship becomes non-linear and quantitation of concentration based on uncorrected SI values will yield erroneous results. This is often referred to as signal saturation in quantitative MRI, not to be confused with the saturation of magnetisation caused by the 90^o^ pulse that generates signal in MRI. It is more helpfully described as a non-linearity effect, generated by a breakdown of the assumption that Gd concentration and SI are linearly related. This is discussed in more detail in the next section.

Extra-vascular, extra-cellular contrast agents are most commonly used in clinical practice. These agents are small enough to leak through the capillaries from the vascular space into the extra-vascular, extra-cellular space but not through cell membranes. It is this property of the contrast agent that enables late gadolinium enhancement of myocardial infarcts where the extravascular, extracellular space is enlarged (see later). Intravascular contrast agents, which stay within the vascular space, are less commonly used but may be preferable for quantitative perfusion imaging as they allow simpler mathematical models to be used for flow quantification as no account needs to be taken of leakage from the vascular space [42].

### Myocardial perfusion imaging

#### Introduction

Myocardial perfusion imaging assesses the blood supply to the myocardium and plays an increasing role in the diagnosis of ischemic heart disease [43]. In this section dynamic contrast enhanced MRI (DCE-MRI) is introduced and the challenging requirements for performing it in the heart are described with reference to pulse sequences described in previous sections. A discussion of the necessary trade-offs that should be considered when designing a perfusion imaging protocol is given. Finally quantification of myocardial blood flow (MBF) from perfusion DCE-MRI datasets is discussed considering the further imaging constraints required for this purpose.

### Dynamic Contrast Enhanced MRI (DCE-MRI)

**Figure 9:**
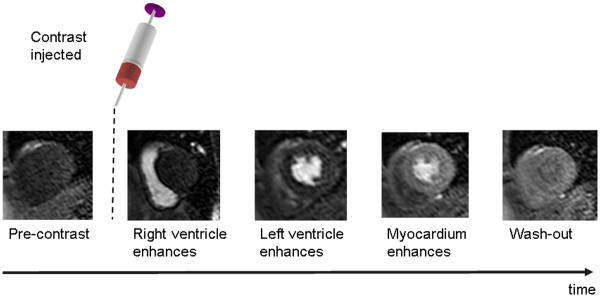
**Dynamic contrast enhanced cardiac perfusion imaging.** Contrast agent is injected intravenously whilst multiple images of the heart are acquired to create a movie showing the contrast agent passing through the heart. Contrast agent can be seen as signal enhancement in the right ventricular cavity (RV) followed by the left ventricular cavity (LV) and more gradually in the myocardium, before finally washing out.

In order to assess myocardial perfusion, blood passing into the myocardium needs to alter image signal intensity so that areas of reduced perfusion can be detected. This is typically achieved using a signal enhancing contrast agent. The contrast agent is injected intravenously whilst multiple images of the heart in the same anatomical position and the same point in the cardiac cycle are acquired in successive heart beats. Typically short-axis images are acquired but a long axis image is also sometimes additionally acquired in order to cover the apex of the heart. In general the acquisition of a dynamic series of MR images during the passage of contrast agent through the body is known as dynamic contrast enhanced MRI (DCE-MRI) [44]. As DCE-MRI generates a movie showing the passage of contrast agent through the body there is a compromise between spatial requirements (i.e. coverage, resolution) at each time point and the temporal requirements (i.e. time interval between successive frames). For myocardial perfusion imaging (MPI), the need to minimise the effects of cardiac motion adds further constraints, making this application of DCE-MRI one of the most challenging. In the ideal case, the dynamic series of images, when viewed as a movie, should be able to demonstrate a motion-free cross-section of cardiac tissue whose signal intensity rises and falls over time as the bolus of contrast agent passes through the myocardial tissue (Figure 9). 

The essential requirements of a DCE-MRI cardiac perfusion imaging sequence can therefore be summarised as follows: All data for multiple images must be acquired within a single heart beat and the effects of cardiac and respiratory motion must be minimised. In addition the image contrast must be T1-weighted to maximise the effect of the contrast agent on image signal intensity. In order to fulfil these requirements, the choice of pulse sequence, method of contrast generation and approaches to minimise motion effects must be carefully considered.

### Choice of acquisition pulse sequence

**Figure 10:**
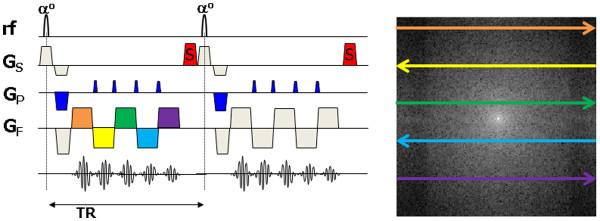
**Pulse sequence diagram for hybrid Echo planar imaging (EPI).** In echo planar imaging (EPI) multiple lines of k-space are rapidly acquired following a single rf excitation pulse. The slope of the frequency encoding gradient is rapidly alternated, generating a train of gradient echoes. A phase encoding gradient ‘blip’ is applied between each frequency encoding gradient to ensure each gradient echo fills a different line of k-space. Single-shot EPI acquires all the lines of k-space following a single rf pulse. More commonly in cardiac MR applications, a hybrid or segmented EPI approach is used where multiple rf pulses are applied each followed by a shorter echo train. In this example, an echo train length (ETL) = 5 is used.

In DCE-MRI the image appearance changes significantly between contiguous frames due to the passage of the contrast agent through the heart so multi-shot k-space imaging strategies that fill k-space over multiple cardiac cycles [1] are not applicable. Therefore, in order to acquire images quickly, DCE-MRI perfusion imaging is generally performed as a single shot technique with a fast (or turbo) spoiled gradient echo (FGE), balanced steady state free precession (bSSFP), or echo planar imaging (EPI) pulse sequence. All three sequences are fast, having a very short TR partly by virtue of the fact that they avoid the need to wait for remnant transverse magnetization to decay after read-out of the MR signal echo. Both the bSSFP and the FGE sequences are described in more detail in part 1 of this review [1]. The bSSFP sequence ‘rewinds’ the signal de-phased by the applied gradients by applying additional balancing gradients to re-phase the MR signal before each subsequent rf pulse. The remnant transverse magnetization is then superimposed onto the magnetization generated by subsequent rf pulses, generating high signal from fluid and blood. Conversely the FGE sequence uses spoiler gradients to destroy remnant transverse magnetization after each readout. EPI uses rapidly alternating frequency encoding gradients, interspersed by phase encoding pulses, to refocus multiple gradient echoes following a single rf-pulse. Single-shot EPI acquires all of the echoes required to fill k-space in a single echo train, however T2* decay throughout the echo train causes the images to be heavily T2*-weighted, resulting in relatively poor image quality. For cardiac imaging a hybrid-EPI (also known as segmented EPI) approach is typically employed where a number of shorter echo trains are acquired by applying multiple rf pulses (Figure 10). This reduces the detrimental effect of T2* weighting, improving the image quality while maintaining some of the speed advantage provided by the EPI technique. 

Despite numerous comparison studies there is still no consensus on the optimal data acquisition pulse sequence for perfusion imaging. Objective measures of pulse sequence performance include the speed of acquisition, the level of artefact and two commonly-used measures of image quality, signal-to-noise ratio (SNR) and contrast-to-noise ratio (CNR). Specifically, SNR is the ratio of the signal intensity of a particular tissue to the background image intensity in a area where signal is absent, while CNR is the ratio of the signal *difference* between two paricular tissues and the background image intensity. As FGE uses a rf-pulse per read-out line it is less susceptible to fluctuations in k-space, which should theoretically make it less susceptible to ghosting artefacts. Nevertheless, hybrid EPI has been shown practically to be less artefact prone the FGE [45]. Hybrid-EPI is also faster, allowing increases in coverage and resolution. By maintaining steady state transverse magnetisation in bSSFP sequences generate the greatest signal of the three methods [46]. The higher SNR of bSSFP images allow a much higher bandwidth to be selected leading to shorter TE and TR making SSFP a faster sequence than FGE [1]. SSFP has been shown to have better sensitivity for detecting perfusion defects [47], also due to its high image SNR and CNR. However of the three sequences SSFP is the most prone to artefacts [15] caused by off resonance magnetization. It has a greater occurrence of susceptibility artefact and ghosting and is prone to Gibbs ringing in the endocardium due to the increased difference in signal intensity between the blood and the myocardium [16]. Explanations of these artefacts are given elsewhere [48,49]. Due to the large number of causes of artefacts with bSSFP it tends to be the least robust sequence, being both capable of producing high quality images but prone to significant image artefacts. Table 2 summarises the advantages and disadvantages of the three sequences. 

**Table 2:** Comparison of the properties of the pulse sequences most commonly applied to cardiac perfusion DCE-MRI

	**←-More**		**Less -→**
Signal	bSSFP	hybrid EPI + FGE	
Speed	hybrid EPI	SSFP	FGE
Artefact Free	hybrid EPI	FGE	bSSFP

The table compares the main data acquisition sequences used in myocardial perfusion imaging: balanced steady state free precession (bSSFP), hybrid echo planar imaging (EPI) and fast gradient echo (FGE) in terms of their signal strength, acquisition speed and propensity for image artifacts.

### Use of preparation pulses for T1-weighting

DCE-MR images should be T1-weighted in order to maximise the effect of the contrast agent on signal intensity. To reduce acquisition time the FGE and segmented EPI sequences described above employ small flip angles and very short TRs resulting in poor T1-contrast, while the bSSFP sequence using a higher flip angle is weighted by the ratio of T2/T1. For this reason a preparation pulse is applied prior to the read-out pulse sequence with a sufficiently long preparation pulse delay to establish a high T1-contrast before the read-out sequence is employed. Currently perfusion imaging is usually carried out using a saturation recovery preparation pulse as inversion recovery increases the total scan time, and is more vulnerable to R-R variation [15].

### Reduction of cardiac and respiratory motion effects

In perfusion imaging each single-shot image acquisition is acquired quickly enough to avoid the detrimental effect of cardiac motion on each individual image. However, respiratory motion still causes mis-registration between adjacent temporal frames. It is typically dealt with by patient breath-holding as the requirement to acquire a dynamic series of images in contiguous heart beats rules out other methods such as respiratory gating. ECG-triggering is also still required to ensure that data acquisition for each image slice is performed at the same point in the cardiac cycle in successive heart beats so that cardiac motion appears frozen when the images are viewed as a dynamic movie.

**Figure 11:**
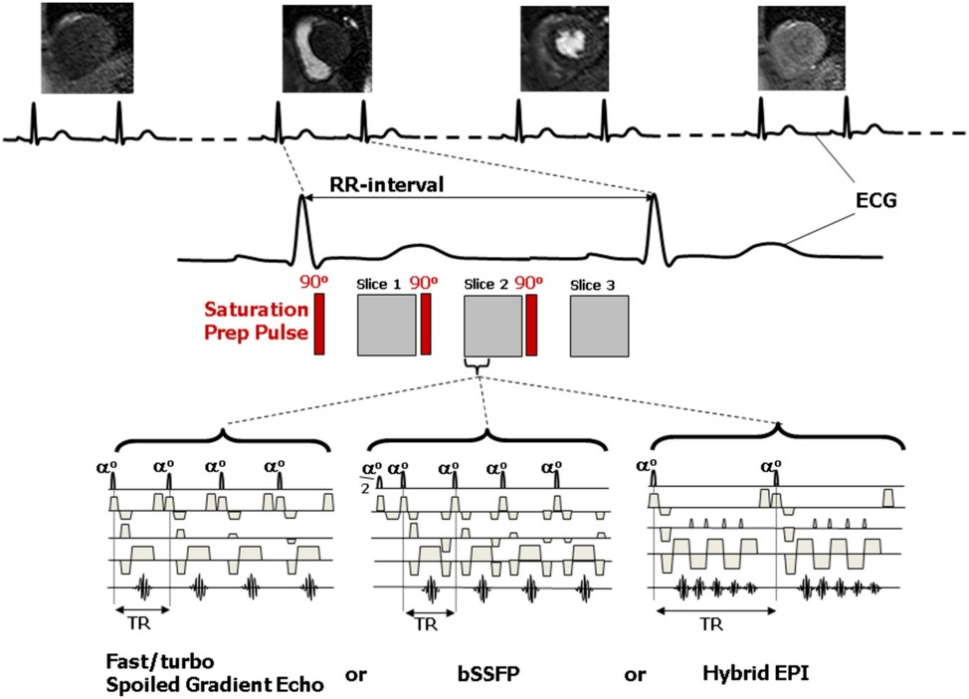
**Choice of imaging pulse sequence for myocardial perfusion MRI.** Diagram illustrating the choices of data acquisition pulse sequence for myocardial perfusion MRI. A saturation recovery preparation pulse is employed to ensure T1-weighting followed after a delay by the image data acquisition pulse sequence. The most common data acquisition pulse sequences used are fast/turbo spoiled gradient echo (FGE), balanced steady state free precession (bSSFP) or hybrid (or segmented) EPI. The number of slices that can be acquired (in this case three) is limited by the R-R interval, the saturation delay and the length of data acquisition period.

Figure 11 summarizes the image data acquisition pulse sequence choices described for perfusion imaging. Once the pulse sequence has been chosen there are a range of further factors to be considered when choosing the imaging parameters for myocardial perfusion DCE-MRI:

For visual analysis of perfusion defects the T1 weighting can be optimised to maximise T1 contrast during the passage of contrast agent through the myocardium. However, if the same image data is to be used for quantitative assessment of myocardial blood flow (MBF), the T1-weighted contrast that is ideal for visual analysis introduces non-linearity in the relationship between the signal intensity and the higher concentrations of the Gd contrast agent found in the blood pool, leading to errors in the quantitation of MBF. For the detection of sub-endocardial perfusion defects there is a requirement to maximise spatial resolution but this increases the acquisition time for each slice which renders the acquisition more prone to cardiac motion and limits the number of slices that can be acquired within a heartbeat, thus limiting coverage of the left ventricle. As the acquisition of sufficient slices to cover the whole heart is desirable, this could be achieved by acquiring an increased number of slices over more than one heart beat, but this increases the time between acquisition of successive images in the dynamic series. For quantitative imaging in particular, it is important to achieve a temporal resolution equal to the acquisition of an image every heart beat. Consideration also needs to be given to the time point in the cardiac cycle that the image data for each is acquired.

These competing factors must be balanced in order to create the ideal pulse sequence for the specific purposes of the study they are being acquired for.

### T1-weighting and TS

T1 weighting is controlled by careful selection of the saturation time (TS). Typically each imaging slice is preceded by a preparation pulse so that there are as many preparation pulses as there are slices in a given RR-interval. To maximise T1-contrast for visualisation of perfusion defects within myocardium longer saturation times should be used. However unnecessarily long TS values take up too much of the RR-interval and limit coverage (by restricting the number of slices that may be acquired) and/or spatial resolution (by limiting the length of the data acquisition per slice). Furthermore if the images are to be used for quantitative imaging shorter TS values are preferable to minimise the non-linearity in the relationship between CA concentration and signal intensity (See later). A typical TS for cardiac perfusion imaging is around 100 ms, but a wide range of values have been used for the reasons described above.

### Factors affecting trigger delay (TD)

**Figure 12:**
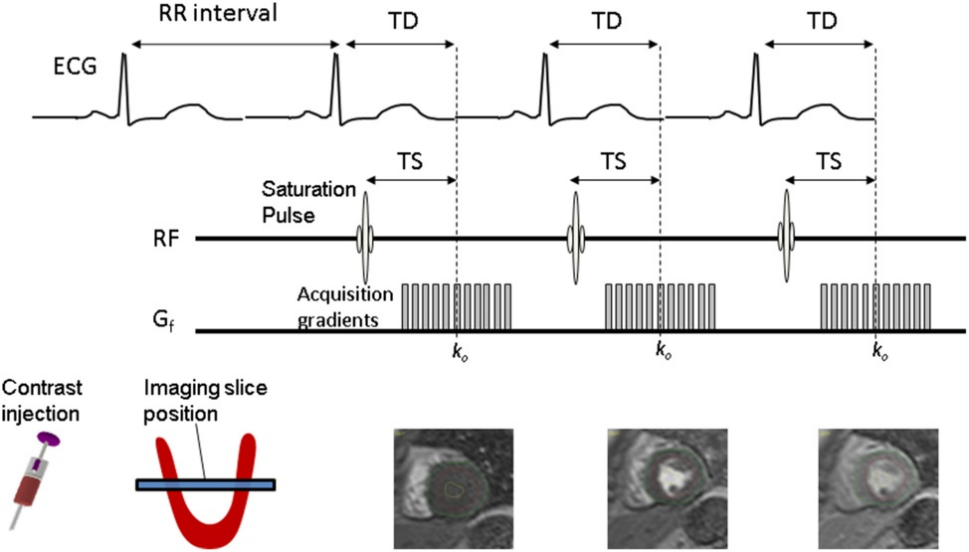
**Trigger Delay and Saturation Time.** The trigger delay (TD) sets the point within the cardiac cycle that the centre of k-space, k_0_, is acquired within each RR-interval . The saturation time (TS) determines the time between the saturation pulse and the centre of k-space, thereby controlling the T1-weighted contrast of the image for a particular image slice.

Image degradation by cardiac motion is dealt with, as far as possible, by limiting the image data acquisition window, so the use of ECG triggering serves only to determine at what phase of the cardiac cycle the heart will be imaged. This is set by the *trigger delay* (TD), which is the time from the ECG R-wave to the time of the acquisition of the central line of k-space, *k*_*o*_, (Figure 12). In a single slice acquisition this can be set to any point of the cardiac cycle. TD does not change with RR interval so if the heart rate increases during imaging the cardiac phase of the image will change during imaging. For long trigger delays, if the heart rate increases substantially then the next R-pulse may occur during the image data acquisition so that part of the image data is acquired from the next heart beat and the true data acquisition for that beat is skipped. The fastest heart motion is during systole and early diastole, thus imaging at mid-diastole should minimise motion artefacts. Conversely there is a preference for imaging in systole if quantitative analysis is foreseen, as the thicker myocardial wall in systole allows larger ROIs within the myocardium and subsequently improved SNR in contrast uptake curves.

### Coverage and resolution

**Figure 13:**
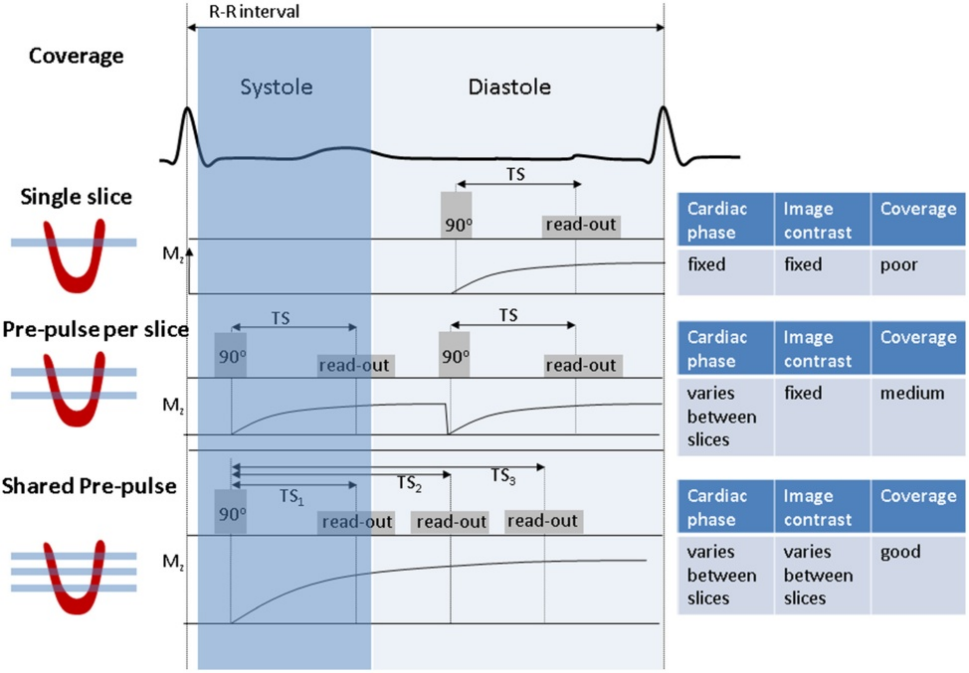
**Choices for myocardial perfusion imaging – prep pulse scheme and number of slices.** With a single slice acquisition per RR interval (top) there is flexible choice for the optimal cardiac phase and T1-weighted image contrast, but poor coverage of the LV. For multiple slice acquisitions, the use of a separate preparation pulse for each slice (centre) allows the same image contrast for each slice (fixed TS) but the two slices are acquired at different cardiac phases due to their different trigger delays and the number of slices is limited (two in this case). Using a pre-pulse shared by all the slice acquisitions (bottom) potentially allows more slices to be acquired (three slices in this case), but leads to each slice having both a different T1-contrast behaviour, as each slice has a different (TS), and a different trigger delay.

Endocardial perfusion defects may occur at any point in the left ventricular wall and so maximising the coverage of the left ventricle is important. The AHA recommend that three short-axis slices are acquired to cover basal, mid and apical regions of the left ventricle [50] and that a spatial resolution of at least 2.5 mm [51,52] is necessary to be able to reliably detect sub-endocardial defects. The achievement of all of these requirements within a single RR-interval is challenging. One approach to increase coverage along the long axis of the left ventricle is to acquire an increased number of slices over 2 RR-intervals, which has the effect of decreasing the temporal resolution of the dynamic series. This is a less desirable option if quantitative assessment of perfusion is required [15,51]. A further alternative is to abandon the requirement that each read-out pulse has a separate preparation pulse [53]. The delay following the saturation pulse, TS, is the longest time delay in the sequence and so the use of a shared saturation pulse applied to all three slices, followed by three slice data acquisitions reduces the acquisition time significantly (Figure 13). However, this approach necessarily results in a different TS value for each slice. Consequentially, the slices have differing image contrast, with the shorter TS value slices being more suited to quantitation as they are less affected by non-linearity effects, while the slices with longer TS values are more suited to visual analysis. 

### Myocardial perfusion image analysis

**Figure 14:**
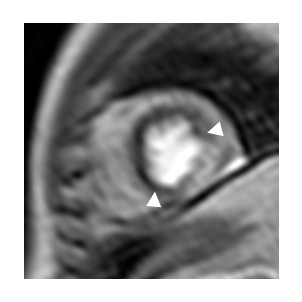
**Myocardial Perfusion defect.** Under stress conditions an area of reduced signal intensity is observed in the septal and anterior walls of the left ventricle, consistent with disease in the coronary arteries supplying these regions. Image courtesy of John Greenwood [43]. (See Additional file 1 for perfusion movie).

The most common method of reporting myocardial perfusion datasets is by visual analysis of the dynamic series of images played as a movie loop. Regions that appear transiently as areas of relative reduced signal intensity are taken to be due to a physiologically significant reduction to myocardial blood flow and recorded as a ‘perfusion defect’ (See Figure 14 and Additional file 1). Alternatively quantitative and semi-quantitative measurements of myocardial blood flow (MBF) have been shown to be useful in the diagnosis of ischemic heart disease [54-56]. 

### Factors relevant to quantitation of myocardial blood flow

**Figure 15:**
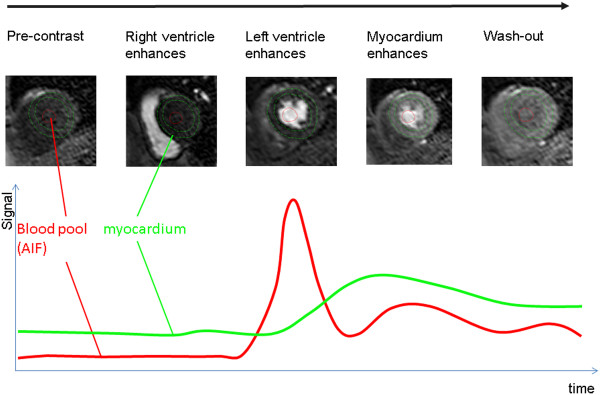
**Quantitative Perfusion Data.** For every image in the dynamic sequence region-of interest contours describing the myocardium and a region in the blood pool are drawn. The mean signal intensity from within each region is plotted for each time point to generate plots of signal intensity versus time to show the increase in signal intensity in both the myocardium (green) and the blood pool (red). The blood pool curve is also often referred to as the arterial input function (AIF). These curves can be analysed together to give an estimate of myocardial blood flow (MBF).

Methods for quantitative analysis of perfusion data have been reviewed in detail elsewhere [51,57]. In brief regions of interest are drawn on each frame of the dynamic series of images to define the myocardium and an area within the left ventricular blood pool. Signal intensities for each of these regions are then plotted at each time point to generate dynamic uptake curves. The blood pool curve is taken to represent the contrast agent passing into the myocardium or the arterial input function (AIF) and the myocardial region represents the contrast agent remaining within the myocardium (Figure 15). These curves are then analysed in order to estimate MBF. 

Semi-quantitative analysis uses a specific property of the time-intensity curve, such as peak height or maximum upslope, as an index of MBF and has been shown to be diagnostically effective [54,55]. Quantitative methods aim to derive an absolute MBF value in ml/min/g by fitting the curves to a mathematical model describing the flow of contrast agent through the myocardium [51,56].

### Non-Linearity effects at high Gd concentrations

**Figure 16:**
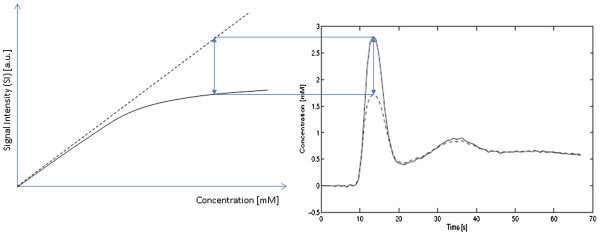
**Non-linearity effects cause errors in MBF.** The left hand graph shows the difference between the assumed linear relationship between signal intensity and Gd concentration (dotted line) and the true relationship (solid line). The right hand graph shows how the non-linear relationship at higher concentrations can propagate into a peak height error in the measured blood pool curve (the arterial input function or AIF) causing an overestimate in MBF.

If perfusion data are to be used for MBF quantitation then an extra consideration becomes relevant in terms of the MR acquisition. The non-linearity in the relationship between gadolinium concentration and signal intensity (SI) must be minimised. Typically for perfusion measurements contrast agent doses do not exceed 0.1 mmol/kg and so the SI to concentration curve has negligible influence from T2 shortening (Figure 8). Figure 16 illustrates how the non-linearity in the relationship between signal intensity and Gd concentration causes blunting of the AIF peak when plotting signal intensity versus time, yielding underestimates in MBF. The degree of.

Nonlinearity depends on the dose and injection rate of the administered contrast agent, the type of MR pulse (EPI, FFE, SSFP) and the saturation time (TS).

Acquisition protocols for quantitative perfusion imaging attempt to optimise these factors to ameliorate the effect of this non-linearity on the MBF estimate. The simplest method is to simply administer a low dose of contrast agent so that the relationship between MR signal intensity and Gd concentration is in the approximately linear region (Figure 16). Contrast agent doses need to be around 0.01 mmol/kg to ensure linearity in the blood pool [58]. These low doses reduce the CNR and SNR of the images rendering visual analysis (still the main-stay of clinical reporting) difficult. The myocardial curve enhances less dramatically than the AIF due to the lower concentration within the myocardium and such low administered doses can reduce the change in signal in the myocardium to such an extent that MBF estimates become significantly affected by image noise, compromising the precision of the MBF estimate. Approaches that try to overcome these issues include mathematical conversion of SI to CA concentration [59,60], dual bolus strategies that repeat the experiment using low contrast and high contrast doses [61,62], and dual sequence strategies that repeat the experiment using two pulse sequences optimised for the blood-pool and myocardial curves [63].

### The dark rim artifact (DRA)

All MR images are susceptible to image artifacts. However in cardiac perfusion imaging the dark rim artefact (DRA) is particularly troublesome as it mimics the very perfusion defects that the investigation is designed to show. DRAs manifest as transient signal voids at the endocardial boundary and are easily mistaken for genuine sub-endocardial perfusion defects. They differ from genuine hypoperfusion events in that they typically last only a few heart beats [64] and they can cause the myocardial signal intensity to drop below the baseline (pre-contrast) signal value [65]. A number of comparison studies have been conducted to investigate which sequences are most prone to DRAs [46,47,66] showing that bSSFP is the acquisition sequence most affected. The cause of a given DRA is difficult to pinpoint as multiple factors have been shown to contribute. Motion during image acquisition can generate abrupt discontinuities in k-space which translate into banding artifacts at tissue boundaries in the image [67]. Magnetic susceptibility effects may also cause DRA due to increase magnetic field distortions around boundaries in the image and temporal changes in magnetic susceptibility on the arrival of contrast agent [47,65]. This effect is most prominent in bSSFP due to its higher sensitivity to changes in magnetic susceptibility which cause local changes to the Larmor frequency. These so-called off-resonance effects become worse at stronger concentrations of contrast agent. The presence of truncation artifact at the high contrast boundary between the blood pool and myocardium is another potential cause of DRA [52]. This is caused by an insufficient content of high spatial frequency data in k-space which means that the Fourier transform is unable to accurately represent true high contrast boundaries in the image. This causes signal variations adjacent to these boundaries that appear as bright and dark bands. This effect becomes worse at higher contrast levels such as with higher bolus concentrations and injection rates, and when using bSSFP as the acquisition sequence. The most reported potential causes and minimization strategies of DRA are summarized in Table 3 although a number other causes have been proposed [15,16]. For a comprehensive and highly informative review of the causes of DRAs see [16]. 

**Table 3:** Causes and solutions of DRA

**Artifact**	**Description**	**Solution**
Truncation	Under-sampling of high frequency data causes signal oscillations at high contrast boundaries	Increase image resolution:
		a) Increase number of phase encoding lines with increased image time.
		b) Use parallel imaging, with decreased SNR
Susceptibility	Increased magnetic field distortions at boundaries and due to arrival of CA cause signal loss and voxel mismapping	a) Avoid bSSFP
		b) Decrease CA concentration
Motion	Discontinuities in k-space due to motion translate to banding at boundaries in the image.	a) Use sequential k-space ordering.
		b) Use parallel imaging

### Delayed imaging after contrast administration

Delayed Gadolinium Enhancement (DGE) imaging is one of the principal CMR techniques. It has been extensively validated, both in animal models and in clinical studies [68-70]. The technique itself is remarkably simple and robust: it involves intravenous administration of gadolinium-based contrast agent followed by the acquisition of T1 weighted images of the myocardium using an inversion recovery technique. DGE imaging is a powerful tool for the assessment of a wide and still expanding range of cardiac pathologies, from acute and chronic myocardial infarction, through inflammatory or infectious myocardial diseases, to cardiac neoplasms [71,72].

Depending on the timing of the acquisition relative to contrast administration, there are two distinct subtypes of the DGE imaging: Early Gadolinium Enhancement (EGE) and Late Gadolinium Enhancement (LGE). These two sub-types of DGE are essentially the same, but the timing of the acquisition (t_a_) following intravenous administration of the contrast agent (0.1 to 0.2 mmol/kg of an extravascular gadolinium chelate) is a distinguishing factor that influences image contrast and provides insights into different aspects of myocardial pathology. Whilst typically t_a_ ~ 5 min post injection is used for EGE, t_a_ > 10 min is used for LGE.

Before describing the nature of temporal changes in longitudinal relaxation time T1 within LV blood and normal and pathological myocardial tissue following intravenous contrast administration, a brief outline of the use of the DGE technique in its primary setting (characterization of myocardial scarring after myocardial infarction) is presented. Temporal changes in T1 and associated changes in signal intensity in inversion recovery T1-weighted images acquired at different times post- injection will then be illustrated using a generalised model of different tissue compartments in DGE studies.

### Viability imaging

The identification of scarred myocardial tissue in patients with acute or chronic myocardial infarction is one of the most important clinical applications of CMR. This method is referred to as “viability imaging” as the absence of scar indicates that the myocardium is viable, i.e. that it retains a capacity to recover contractile function following revascularisation. The information on the location and the extent of myocardial scaring is therefore important for planning coronary artery revascularisation. As the size of scarred myocardium and its location relative to the endocardial border are important predictors of the potential for functional recovery [73], CMR assessment of myocardial viability by DGE has an inherent advantage over other imaging methods (such as PET, SPECT and echocardiography) due to its superior spatial resolution.

In addition to the identification of the location and the extent of scarred tissue by LGE, EGE provides important diagnostic information about the presence and the extent of microvascular obstruction (MVO). MVO is also known as the no-reflow phenomenon [74] and its presence serves as a significant negative predictor of functional recovery post percutaneous coronary intervention (PCI).

**Figure 17:**
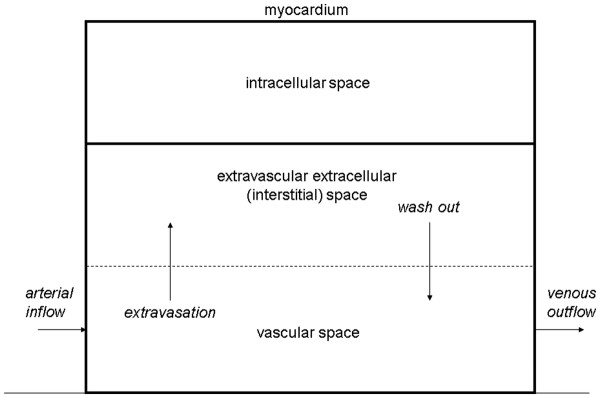
**Schematic illustration of an extracellular Gd- based contrast agent kinetics within the myocardial tissue.** Following intravenous administration, the extracellular Gd-based contrast agent enters the microvascular network of the myocardium via arterial inflow (left). It is extravasated into the interstitial fluid (extravascular extracellular space) and gradually washed out back into the venous outflow (right) and removed from the body via glomerular filtration. Areas with an increased volume of interstitial space will present a larger distribution volume for the incoming contrast agent, but importantly pathological tissues such as myocardial scar, may also display slower extravasation rates as well as delayed re-absorption (wash out) of contrast agent into the vascular space.

CMR viability assessment is currently conducted by collecting T1-weighted inversion recovery images of the myocardium several minutes after a peripheral administration of a contrast agent (gadolinium chelate) that selectively alters tissue relaxation times in proportion to local tissue concentration (see earlier). The local tissue concentration of exogenous contrast agent changes over time following bolus administration and the rate of this change is directly related to several physiological variables. First, the agent needs to reach the region of interest via systemic and peripheral coronary circulation (arterial inflow). Next, it is distributed throughout the myocardial capillary network (Figure 17).

Areas supplied by patent coronary vessels and occupied by a dense capillary network will exhibit strong enhancement of the MR signal during first pass of the contrast agent, as the local concentration of contrast agent carried by blood will cause strong acceleration of longitudinal relaxation (T1 decrease), and consequent pixel intensity increase on T1-weighted images (Figure 8).

Another important process takes place at the same time: extravasation of the contrast agent and its accumulation within the extravascular extracellular (interstitial) fluid. Areas with an increased volume of interstitial space will present a larger distribution volume for the incoming contrast agent, but importantly pathological tissues such as myocardial scar, may also display slower extravasation rates as well as delayed re-absorption (wash out) of contrast agent into the vascular space (Figure 17).

This is why scarred tissue contains high concentrations of contrast agent compared to normal (viable) myocardium several minutes after bolus administration. It is this difference in *delayed* local tissue concentration within the interstitial fluid that gives rise to image contrast on what is generally referred to as delayed gadolinium enhancement CMR: scarred myocardium appears relatively hyperintense compared to surrounding viable myocardium on T1-weighted images. This same principle applies to acute infarction and fibrosis as well as chronic scar with each appearing bright on LGE images.

To maximise the effect of these differences in contrast agent concentration, the image readout needs to be timed to coincide with the point at which the difference between viable and scarred tissue concentration is most pronounced and optimal T1-weighting needs to be achieved to translate these concentration differences into strong image contrast.

The following sections describe how the changes in contrast agent concentration affect T1 values of LV blood, normal myocardium, scar and MVO, and how T1 values change over time. They will also describe the effect those changes have on MR signal intensity, and how to achieve image contrast suitable for the delineation of scar and MVO.

### Time-related changes in T1

Although differences in native T1 values between normal myocardium and regions affected by different pathological processes exist in the pre-contrast state, they tend to be subtle and difficult to measure using currently available techniques. Following the administration of an intravenous contrast agent various tissues experience different degrees of T1 shortening, and the assessment of these *changes* in native tissue T1 enables us to distinguish between normal myocardium and various pathologies.

The differences in the temporal pattern of the return to the pre-contrast state, and the extent of the SI changes, give a very powerful (if indirect) measure of the local tissue haemodynamics and water content. Of course, systemic factors could potentially obscure this process of differentiation and must therefore be taken into account.

Although the exact mechanisms behind the complex contrast patterns that characterise EGE and LGE in particular are still not fully understood, it is helpful to try to explain them in the context of two distinct phases of extracellular contrast agent pharmacokinetics, namely early access phase (EGE) and late distribution phase (LGE). To illustrate the principal contrast-inducing process (contrast agent induced temporal T1 changes), a set of measurements adopted from a study by Klein et al. [75] will be used.

Following bolus injection of T1-shortening contrast agent, a transient sharp increase in the blood signal (arterial input function peak) is followed by several oscillations (recirculation peaks) after which a gradual approach to equilibrium occurs. In the equilibrium the concentration of contrast agent in the circulating blood is in balance with the concentration in the interstitial compartment (Figure 17).

**Figure 18:**
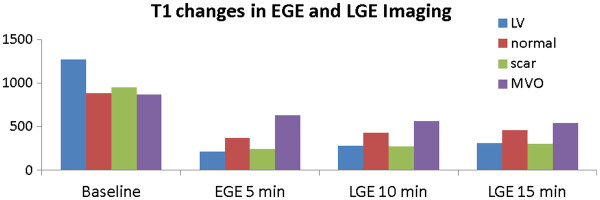
**Time-course of T1 for EGE and LGE.** This shows the time-course of the changes in longitudinal relaxation time T1 in LV and myocardium following administration of 0.2 mmol/kg Gd-DTPA. T1 values are adopted from the data measured in a study by Klein et al. [75]. In EGE imaging (t_a_ ~ 5 min post-injection), all tissues apart from MVO experience a significant T1 shortening (second panel from the left). In areas occupied by MVO, a very modest amount of contrast agent is present at 5 min post-injection, and the T1 value within the MVO is high compared to the other three compartments at this time point. The rate of recovery towards the baseline (pre-contrast) T1 value reflects the washout of contrast from individual compartments. Whilst normal myocardium and LV blood T1 values continue to rise between 5-15 min post-injection, scar tissue still maintains low T1 values, due to delayed extravasation and accumulation of contrast agent within an enlarged interstitial water compartment. The low values of T1 may further be maintained by the slow washout kinetics. In MVO, T1 values may continue to decrease, as the areas occupied by MVO may receive contrast agent via passive diffusion from the neighbouring scar.

The first phase (access phase) is dominated by the differences in microvascular blood supply – the existence and “quality” of the myocardial capillary network. In areas where this network is non-existent or disrupted, contrast agent will have limited access and therefore little or no impact on signal intensity. Therefore, following intravenous injection, contrast agent carried by the blood will shorten the T1 values in the areas that are normally perfused, but will have little or no effect on areas with disrupted or non-existent blood supply, such as microvascular obstruction or thrombus. In EGE imaging (t_a_ ~ 5 min post-injection), all tissues apart from MVO experience a significant T1 shortening (Figure 18, second panel from the left).

In areas occupied by MVO, a very modest amount of contrast agent is present at 5 min post-injection, and the T1 value within the MVO is high compared to the other three compartments at this time point.

The rate of recovery towards the baseline (pre-contrast) T1 value reflects the washout of contrast from individual compartments. Whilst normal myocardium and LV blood T1 values continue to rise between 5-15 min post-injection, scar tissue still maintains low T1 values, due to delayed extravasation and accumulation of contrast agent within an enlarged interstitial water compartment (Figure 18). The low values of T1 may further be maintained by the slow washout kinetics [76].

In MVO, T1 values may continue to decrease, as the areas occupied by MVO may receive contrast agent via passive diffusion from the neighbouring scar [77].

### Pulse sequences for EGE and LGE

In CMR perfusion imaging signal acquisition needs to be performed during the first pass of the contrast agent through the myocardial capillary bed. The contrast agent concentration changes very rapidly during this time, thus requiring a dynamic imaging technique using an ultra-fast single shot acquisition pulse sequence (see previous section on perfusion imaging). In DGE the signal collection is performed during the equilibrium phase of contrast agent kinetics when the concentration of contrast agent changes relatively slowly and image data acquisition over several heart beats using a cardiac-triggered, multi-shot imaging pulse sequence [1] is therefore possible. This means that high spatial resolution DGE images can be acquired, a factor that is important for accurate delineation of scar size and especially its transmural extent.

**Figure 19:**
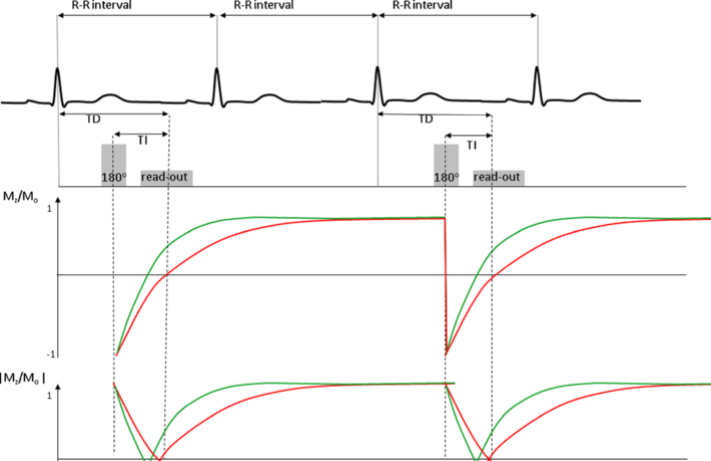
**Pulse sequence diagram for IR-GRE.** Pulse sequence diagram representing two consecutive segments of an IR-FGE sequence used in early and late Gadolinium enhancement imaging. In a typical breath hold acquisition of a single DGE slice, 8–10 segments are acquired to fill ~192 -240 lines of k-space. The changes in M_z_/M_0_ are illustrated in the middle panel, with green and red lines representing M_z_/M_0_ in short and long T1 regions. The bottom panel illustrates the changes in signal intensity in the modulus MR images (SI ~ |M_z_/M_0_|). For each shot, several lines of k-space are filled within an image data acquisition window typically limited to 150-200 ms, with a trigger delay corresponding to mid-diastole to minimise the effects of cardiac motion.

DGE images are typically acquired using an inversion recovery, segmented, fast (or turbo) gradient echo sequence (IR-FGE) [1]. For each shot, several lines of k-space are filled within an image data acquisition window typically limited to 150-200 ms, with a trigger delay corresponding to mid-diastole to minimise the effects of cardiac motion (Figure 19). The cardiac triggering parameters are set to acquire image data in every second heart beat, to allow sufficient signal recovery over 2 RR intervals. This ensures almost complete recovery of the z-magnetisation for all tissues with T1 shorter than approximately 0.4 * RR (i.e. 400 ms for HR = 60 bpm). In cases of tachychardia or bradycardia, data acquisition can be spaced in every third or every RR interval, respectively. The main physiological limitation in the choice of imaging parameters for this sequence is the length of the breath hold that a patient can comfortably sustain. This determines the number of RR intervals that can be used for signal collection, depending on patient’s HR. Typically 8–10 shots are acquired within 16–20 RR intervals, and the number of lines of k-space that are acquired within each individual shot will determine the resulting spatial resolution for a given field of view. The number of lines of k-space in each shot is determined by the operator as the number of views per segment (GE), turbofactor (Philips) or number of segments (Siemens). Increasing this parameter shortens the breath-hold period at the expense of a longer data acquisition window, which may lead to motion induced blurring. 

With the readout timing limitation (150 to 200 ms) and a typical TR of 5-10 ms, 15 to 40 lines of k-space can be acquired within a single data acquisition window. A typical acquisition matrix is 240 x 240, which yields DGE images with a voxel size of 1.75 mm, for a FOV of 420 mm.

Each image data acquisition is preceded by a non-slice-selective inversion recovery preparation pulse to provide T1-weighting. As well as producing strong T1 contrast, the inversion recovery (IR) technique has the additional benefit that is it is possible to suppress the signal from tissue of a particular T1. Careful choice of the time delay after inversion, TI, allows the optimisation of contrast between tissues with different contrast agent concentrations including selective suppression of one of these tissues. The TI value is either fixed (~440 ms, as in EGE) or determined empirically for LGE applications (see next section).

With this choice of timing for the inversion pulse (Figure 19), the z-magnetisation for the tissue with longer T1 (red line) will be close to zero at the time of the readout. The voxels occupied by tissues with this T1 value will therefore have very low SI on MR images (i.e. this T1 value will be “suppressed” or “nulled”).

In a typical LGE exam, 10–12 breath hold slices are acquired in short axis orientation, followed by long axis and 4-chamber views where clinically indicated. The choice of TI needs to be periodically updated if the examination is prolonged to ensure optimal nulling of the normal myocardial tissue, as the T1 of the normal myocardium will continue to increase gradually during this time.

### Optimising T1 contrast in EGE an LGE

The image contrast between different tissue compartments is controlled by choosing an appropriate inversion time, TI to highlight the differences in their T1 values. This implicitly highlights the differences between time-variant concentrations of Gd-DTPA in individual tissue compartments, as described in the earlier section. As LV blood pool and scar have very similar T1 values between 5–15 minutes post contrast, their recovery curves closely follow each other. Separation of scar and blood pool signal is therefore difficult to achieve. However, an appropriate choice of TI will highlight the differences between MVO and normal myocardium in EGE. In LGE, the TI can be fine-tuned to emphasize the differences in T1 of normal myocardium and scar.

**Figure 20:**
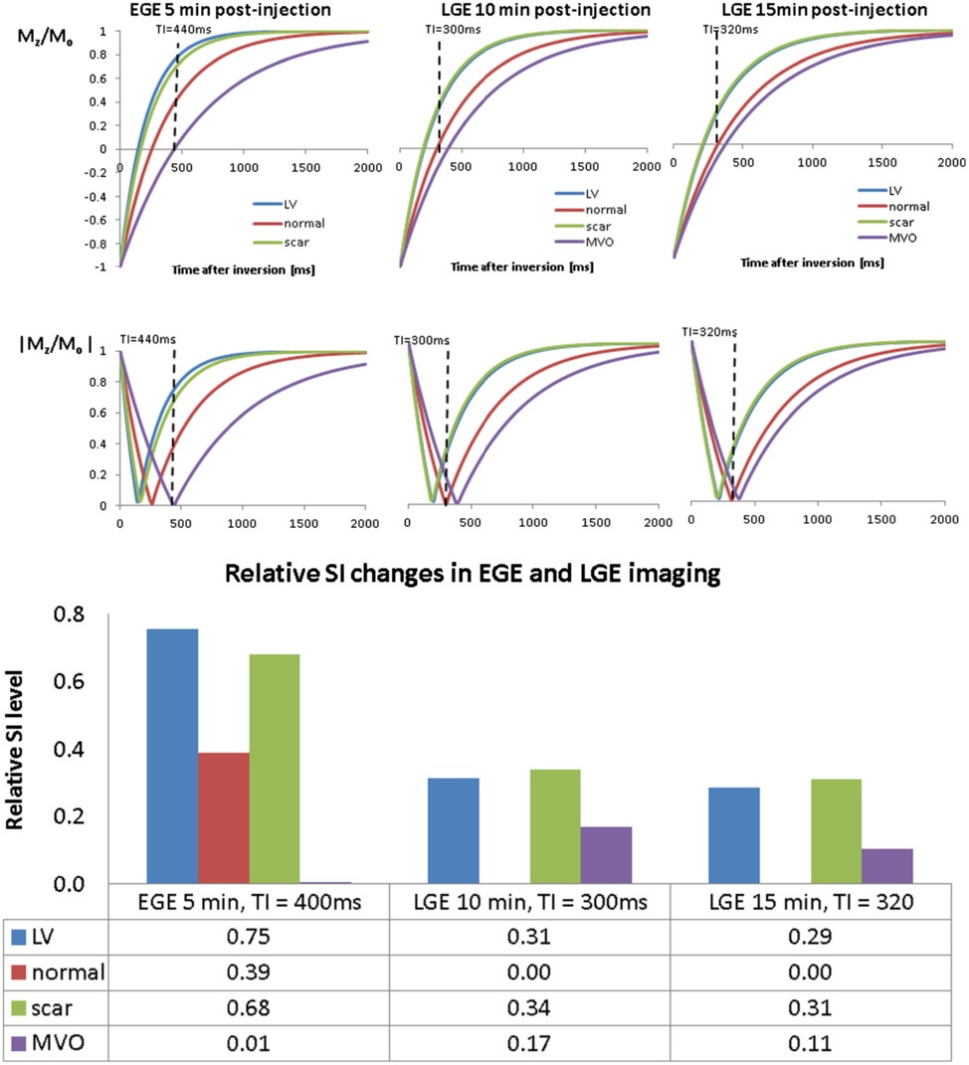
**Inversion recovery curves at 5-15mins post-injection.** Inversion recovery curves at 5, 10 and 15 minutes post-injection and relative signal levels obtained by choosing TI values to suppress signal form MVO (purple curves) at 5 minutes post injection and normal myocardium signal (red curves) at 10 and 15 minutes post-injection. Curves are shown of both the z-magnetisation, M_z_ expressed as a fraction of the magnetisation at equilibrium, M_o_ (M_z_/M_o_) and the modulus of this value, (|M_z_/M_o_|), representative of the actual displayed signal intensity. In EGE, the difference between T1 in the MVO compartment and surrounding scar and normal myocardium is emphasized by using a long TI which minimises the signal from the MVO. A TI of 440 ms will null the signal from MVO (T1 = 640 ms at 5 minutes post-injection). With this choice of TI, surrounding scar and normal myocardium appear bright, thus enabling identification of MVO (left panel). At 10 and 15 minutes post-injection, the differences in T1 between normal myocardium and scar begin to emerge due to delayed accumulation of contrast in the enlarged interstitial space of the scar. To maximise contrast between scar and normal myocardium, a TI of 300 ms is chosen to null the signal from normal myocardium at 10 min LGE images (middle panel). As T1 values continue to change between 10 and 15 minutes post injection, the optimal nulling time rises to 320 ms in at 15 minutes post-injection for the example presented above (right panel).

Figure 20 illustrates inversion recovery curves in four tissue compartments using T1 values presented in the section on time-related changes in T1 (Figure 18). This figure shows how temporal changes in T1 affect the patterns of SI recovery to equilibrium and shows the importance of choosing the optimal TI to achieve desired image contrast.

In EGE, the difference between T1 in the MVO compartment and surrounding scar and normal myocardium is emphasized by using a long TI which minimises the signal from the MVO. In the example presented in Figure 18, a TI of 440 ms will null the signal from MVO (T1 = 640 ms at 5 minutes post-injection). With this choice of TI, surrounding scar and normal myocardium appear bright, thus enabling identification of MVO (Figure 20, left panel).

At 10 and 15 minutes post-injection, the differences in T1 between normal myocardium and scar begin to emerge due to delayed accumulation of contrast in the enlarged interstitial space of the scar. To maximise contrast between scar and normal myocardium, a TI of 300 ms is chosen to null the signal from normal myocardium at 10 min LGE images (Figure 20, middle panel). As T1 values continue to change between 10 and 15 minutes post injection, the optimal nulling time rises to 320 ms in at 15 minutes post-injection for the example presented above (Figure 20, right panel).

Overall signal levels continue to decrease during the late enhancement phase as LV and normal myocardium continue their return to their pre-contrast (long) T1 values.

**Figure 21:**
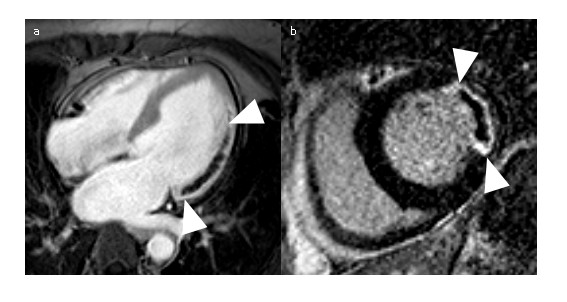
**EGE images. a**) EGE image acquired in the 4-chamber orientation demonstrating a large area of microvascular obstruction in the lateral wall associated with transmural infarction that extends from base almost to the apex (arrowheads). **b**) Short axis LGE image depicts a transmural scar (arrowheads) that envelops an area of MVO in the lateral wall. Images courtesy of Dr J. Greenwood, MCRC, University of Leeds.

In contrast to EGE, where a fixed TI (~440 ms) normally yields very good contrast between the MVO and the surrounding scar and normal myocardium (Figure 21), the TI value needs to be determined empirically in LGE studies. The LGE acquisition is therefore preceded by a TI-scout module, described in the following section.

### Selection of TI for optimal LGE imaging – the ‘TI scout’

The main practical difference in the acquisition of EGE and LGE is that LGE is preceded by a TI-sweep Look Locker module [78], designed to find the optimal inversion time TI to null the signal from the normal myocardium and maximize the contrast between normal myocardium and scar tissue.

The exact value of this parameter will depend on the individual haemodynamics and systemic water compartmentalisation that will define global contrast agent kinetics in individual patients. The optimal TI will also change over time, as T1 values in all four compartments continue to change. Whilst LV blood and normal myocardium T1 values continue their recovery towards their baseline (pre-contrast values) during this late enhancement phase, T1 values in scar tissue and MVO follow a different pharmacokinetic path determined by the volume of the interstitial space, wash-in and wash-out rates and, in the case of MVO, passive diffusion of contrast from the surrounding scar tissue.

For example, the optimal TI that will suppress the signal from normal myocardium will be 300 ms at 10 minutes post-injection in the example presented in Figure 18, but at 15 minutes post-injection, the optimal TI will be 320 ms (Figure 20).

As exact values of T1 in individual tissue compartments are not known ahead of IR-GRE acquisition, optimal values of TI need to be determined empirically. In the T1-sweep Look Locker module, TI values are changed incrementally before each image readout. The resulting images display different contrast between the four compartments, and allow the operator to identify the TI which best minimises the signal from normal myocardium.

**Figure 22:**
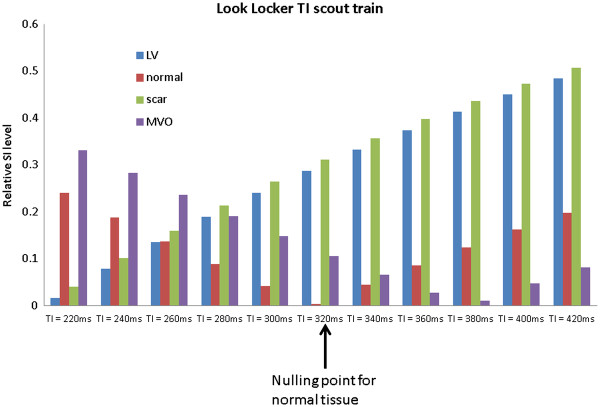
**Look-Locker TI scout train.** This figure shows the relative signal intensities for the LV blood pool, normal myocardium, scar tissue and MVO for the images acquired at different TI values using a TI scout (Look-Locker technique). The image where the normal myocardium appears darkest is identified as the optimal TI time. In this example, TI of 320 ms nulls the signal from the normal myocardium.

In the example presented in Figure 22, relative signal intensities in four tissue compartments are presented as a function of incrementally changing TI. The image where normal myocardium appears darkest, identifies the optimal inversion time (320 ms in the example used here).

### Alternative DGE pulse sequences

Although the standard IR-FGE sequence combined with a modulus image reconstruction is still the most widely used in clinical practice, there are other approaches that aim to address different shortcomings of this standard approach.

**Figure 23:**
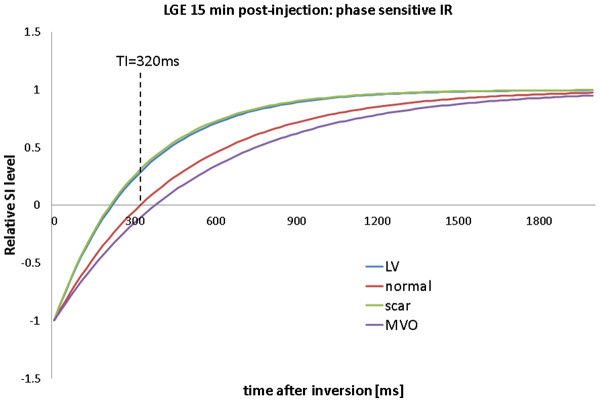
**Phase sensitive inversion recovery sequence.** By using a phase sensitive reconstruction, the sign of magnetisation is reflected in the displayed signal intensity. The signal intensity is mapped onto a grey scale showing values of zero at the centre of the grey scale, positive values as higher pixel intensities (towards white) and negative values as lower pixel intensities (towards black). Using this method, the effect of a small error in the choice of optimal TI is reduced. The normal myocardial signal can be suppressed by careful windowing of greyscale.

As IR-FGE contrast using modulus reconstruction is very sensitive to the choice of TI, and small variations in TI can cause different distributions of signal intensities in typical modulus images (Figure 20), a pulse sequence and image reconstruction which can take account of the sign of the z-magnetisation at the time of data acquisition was developed to alleviate this problem and increase the dynamic range of IR signal intensities [79]. This approach is called Phase Sensitive Inversion Recovery (PSIR) and the effect that it is designed to produce is illustrated in Figure 23. 

As the polarity of the signal is restored, relative signal levels between normal myocardium and scar can be captured over a wide range of inversion times, and precise estimation of TI via a TI Scout module is no longer necessary.

**Figure 24:**
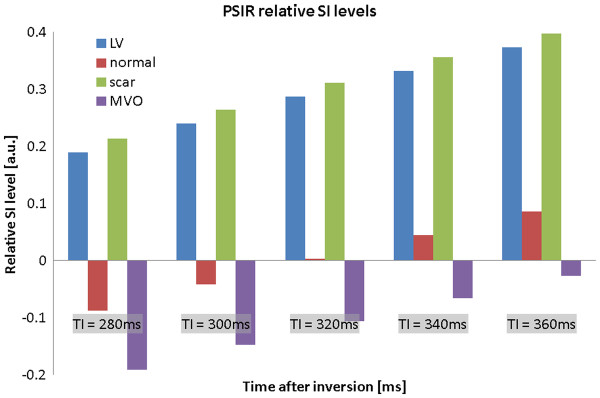
**Relative SI levels in Phase Sensitive IR sequence.** By restoring signal polarity (Figure 23), normal myocardial signal remains markedly lower than scar, even if the null point is underestimated by 40 ms. Note that negative signal intensities correspond to tissues who’s longitudinal magnetization has not recovered past M_z_ = 0 at time TI. For image display purposes, tissues with zero magnetisation values are mapped onto the middle of the image pixel intensity range. Tissues with a positive magnetisation are then displayed with image pixel intensities increasing from this middle value, while tissues with a negative magnetisation are displayed with images pixel intensities decreasing from this middle value. ‘Nulling’ the normal myocardium can then be achieved retrospectively by adjusting the window and level on the viewing console and there is no longer an ambiguity between tissues with positive and negative magnetisation values.

As can be seen in Figure 23, once signal phase is restored, normal myocardium will continue to have low signal intensity compared to scar over a wide range of TI values. Relative signal levels for TI ranging from 280 ms through 320 ms (true null point for normal myocardium), to 360 ms are illustrated in Figure 24 below.

By collecting the IR prepared signal in a 3D mode, significant savings in imaging time can be achieved [80,81] However, the required readout window is longer compared to 2D and the spatial resolution is significantly reduced too. To offset these drawbacks, navigated free-breathing approaches have been proposed [82,83].

In addition to these efforts directed at collecting DGE images in a more time-efficient manner, there is an ongoing research into alternative signal preparation schemes that would improve the contrast between scarred and normal myocardium, as well as scar and blood [84,85]. With a wider use of parallel imaging, further refinements in both DGE preparation and readout are likely to emerge in the coming years [7].

### Magnetic Resonance Angiography (MRA)

In this section, three different approaches to performing MR angiography will be described. First, the most common approach, known as contrast enhanced MR angiography (CE-MRA) involves the use of a Gadolinium-based contrast agent to delineate the vessel lumen and is an extremely fast and reliable method for imaging the vasculature outside the heart. Due to concerns relating to the use of contrast agents in patients with poor renal function, there has been a resurgence of non-contrast MRA techniques to image vasculature outside the heart. The exact approach is dependent upon the vessel to be imaged, but this section provides a brief overview of the currently available methods. For all of the above MRA methods, image degradation caused by respiratory motion within the thorax and abdominal regions is eliminated by performing the acquisition during patient breath-holding. However this limits the acquisition time and hence the spatial resolution of the method. For imaging of the coronary arteries, their relatively small diameter, together with significant motion during the cardiac cycle, make the standard MRA techniques ineffective. The final part of this section describes the most common approach to coronary MRA, respiratory navigator-gated 3D coronary angiography. This is performed without the use of contrast agent over several minutes to achieve higher spatial resolution. Respiratory motion is compensated for by accurately gating the acquisition with respect to the respiratory cycle by using special navigator rf pulses to accurately track diaphragm position, while the influence of cardiac motion is removed by using ECG triggering to synchronise the data acquisition to mid-diastole.

### Contrast-enhanced MRA (CE-MRA): the basic principles

**Figure 25:**
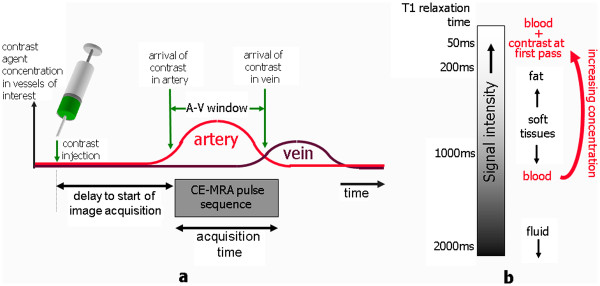
**This schematic diagram illustrates the key principles of CE-MRA.** The diagram in (**a**) shows the relative timing of the key events in a CE-MRA acquisition. Following the intravenous injection of contrast agent into a subject, there is a delay as the contrast bolus travels through the right and left sides of the heart before entering the arterial system. The red and black curves show the variation of concentration of contrast agent in the arteries and veins within the region of interest over time. Optimum image quality is achieved if the image data acquisition is timed to commence after the arrival of the contrast bolus within the arterial system of interest and is completed before the arrival of contrast in the corresponding venous system (sometimes referred to as the A-V window). The schematic diagram in (**b**) shows qualitatively how signal intensity relates to tissue type for a typical T1-weighted MRA pulse sequence. Indicative values for T1 relaxation time are also shown. Without the introduction of contrast agent, fat tissue would have the brightest signal in the image and blood within the vessel lumen would not be visible. The introduction of contrast agent into the blood reduces its T1 relaxation time and increases the signal intensity. The maximum concentration during first pass of the contrast bolus through the arterial system must be sufficient to reduce the T1 of blood to significantly below that of fat, so that the arterial vessel lumen yields the highest pixel intensities on the image.

The basic principle behind CE-MRA is essentially the same as that of conventional x-ray angiography: A bolus of contrast medium is injected intravenously and imaging is performed during the first passage of the bolus through the vessels of interest [86,87]. As it is desirable to maximise the contrast generated by the presence of the contrast medium, the aim is to maximise the signal enhancing effect of contrast medium in the vessels of interest, while minimising the concentration in the surrounding tissue and unwanted vessels. The CE-MRA pulse sequence must therefore be fast enough to be completed within a limited time window during maximum arterial enhancement and before venous enhancement, sometimes referred to as the arterio-venous window (AV) window (Figure 25a). 

In order to perform CE-MRA successfully it is important to understand the practical implications of the above requirements. The following sections will explain the basis of choosing the volume and duration of the bolus injection, timing the image acquisition to coincide with the first pass phase of the contrast medium and selecting a suitable pulse sequence and its parameters to provide a sufficiently rapid data acquisition whilst maintaining good contrast, 3D coverage and spatial resolution.

### The basis of image contrast in contrast-enhanced MRA (CE-MRA)

**Figure 26:**
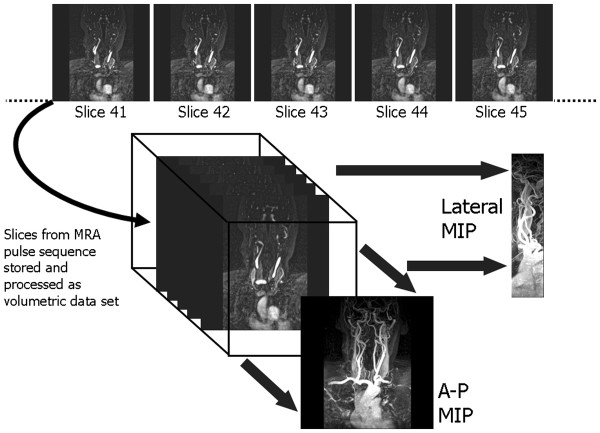
**Maximum Intensity projection (MIP).** This diagram shows an overview of how projection images can be produced from multiple slice MRA image data. For 2D acquisitions, multiple thin overlapping slices are acquired. For 3D acquisitions, the selected volume is encoded and partitioned into multiple thin, contiguous slices. The MRA technique and its acquisition parameters are chosen to produce a signal intensity from the vessels of interest that is significantly higher than that of the background tissues. The maximum intensity projection (MIP) is produced using computer processing software that traces parallel paths through the data in the direction of the intended projection image, records the maximum pixel intensity encountered and stores this onto the projected image. The lateral and A-P views shown are typical of those produced automatically by the software immediately following image reconstruction. It is usually possible to also prescribe automated reconstruction of customized MIPs at any arbitrary angle. Further MIPs can also be produced retrospectively on a workstation. A disadvantage of the automated MIPs shown is that they often contain overlapping vessels and a relatively high signal from background tissue. This can be removed with interactive post-processing software so the MIP algorithm is only applied to a selected volume of data, sometimes referred to as targeted MIP.

CE-MRA relies on achieving a difference between the T1 relaxation value of the blood containing the contrast media and that of the surrounding tissues that results in the vessels of interest appearing as the brightest structures on the images. If the difference between the T1 of the blood and that of the next brightest structure on the images, (usually fat), is sufficiently great, then the fat and the other tissues can be eliminated by windowing. This then allows simple post-processing methods [88], including maximum intensity projection (Figure 26 and Additional file 2) to provide images of the vascular tree with ease. In non-contrast imaging, fat has the shortest T1 value of all the tissues (in the range 170-230 ms depending on field strength). For standard T1 weighted imaging it therefore has the highest signal of all the tissues in the image (Figure 25b). Since, for CE-MRA, a much higher signal is required from the vessels relative to the other tissues, the concentration of contrast media achieved during first pass through the vessels must be sufficient to reduce the T1 value of the blood to well below that of fat. Furthermore, as very short TR values are required to achieve the fast scan times, it is necessary to lower the T1 value of blood sufficiently to avoid undue saturation of the signal within the vessels. Typically, for CE-MRA, it is desirable to achieve T1 values of the order of 50 ms or less. 

Conventional T1-weighted anatomical imaging using spin echo or spoiled gradient echo pulse sequences is achieved using a short TR and a short TE [1]. For both these techniques, the TR is chosen so that there is good contrast between most of the tissues, and most of the tissues (apart from fluid) will contribute a reasonable signal. For CE-MRA, much shorter TR values are used. In this case, the signal from all of the tissues, including fat, is significantly reduced. High signal intensities only exist where the contrast medium is present at sufficiently high concentrations.

The concentration of contrast agent during first pass, c_first pass_ required to reduce the T1 of blood to around 50 ms can be calculated using the relationship described in the early section on contrast agents, which relates T1 relaxation rate to the concentration of contrast agent of a given relaxivity, r_1_. For most currently available gadolinium-based contrast media, the T1 relaxivity is approximately 0.004 ms^-1^ mM^-1^ for field strengths of 1.5 T. Estimating the T1 of blood without contrast agent as 1200 ms, the required concentration can be estimated to be around 5 milliMolar (mM).

The original concentration of the contrast medium (from the bottle) depends on the particular contrast agent used, but a typical value might be 0.5 mmol/ml. (=0.5 mol/l = 0.5 Molar concentration), which is about 100 times too high. Once injected, however, the initially high concentration of contrast medium within the bolus becomes diluted as it travels from the injection site by gradual mixing with the blood pool as it travel first though the right side of the heart, the pulmonary circulation and then the left side of the heart.

By making some assumptions about the physiology of the patient and that the contrast agent mixes evenly with the blood pool during the first pass stage, it is possible to estimate how mixing with the blood pool affects concentration of the contrast medium in the arterial system during the first pass for a given injection rate as follows [89]:

$$C_{\text{first pass}}=C_{\text{bottle}}\cdot \left(\text{Injection rate}/\text{Cardiac Output}\right)$$

where C_bottle_ is the original concentration of the contrast medium (from the bottle). For a typical person the cardiac output is around 6 litres/minute, which is equal to 100 ml/s. Using the above relationship to achieve the desired dilution of C_first pass_ / C_bottle_ = 1/100, the required injection rate must be around 1 ml/s. In clinical practice, injection rates of between 1–5 ml/s are in use. An increase in injection rate results in a higher concentration of contrast agent during the first pass, further reducing the T1 of blood and increasing the signal intensity within the vessels. The duration of injection will determine the duration of the contrast bolus. Ideally, this should match the acquisition time of the MR pulse sequence. The injection rate and the duration of injection together determine the volume of contrast agent delivered and therefore the dose. Each of these parameters needs to be carefully balanced to avoid giving the patient unnecessarily large doses of contrast agent, while maximising signal intensity. There is clearly an advantage in choosing acquisition techniques that can achieve shorter acquisition times, leading to a shorter duration of injection and lower contrast dose without compromising the injection rate.

There is also an effect of the contrast medium upon the T2 relaxation time, which is determined by the T2 relaxivity, r_2_. For field strengths from 0.5 to 1.5 Tesla, r_2_ is approximately 0.006 ms^-1^ mM^-1^. If it is assumed that the T2 of blood without the contrast medium is approximately 200 ms, then by a similar calculation, the observed T2 of the arterial blood during first pass is approximately 28 ms. The T2 of the blood is therefore also considerably shortened, but not by the same proportion as the T1. To minimise any signal loss caused by the reduction of the T2 value of the blood, the echo time of the pulse sequence must be kept as short as possible. As a rule of thumb, provided that the T2 is greater than two times the TE, signal loss due to T2 effects will not be significant, except in regions where the concentration is closer to the original concentration within the injected vein. This is often seen as signal drop-out within the vein close to the site of injection.

### The CE-MRA pulse sequence

**Figure 27:**
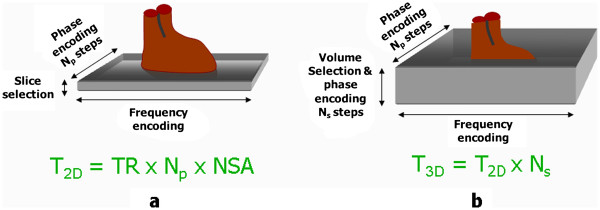
**Comparison of 2D and 3D image acquisition.** This diagram shows the key differences between 2D and 3D image acquisitions. For 2D acquisitions (**a**) a slice is selectively excited, and the MR signal is encoded in two dimensions by phase encoding and frequency encoding. For conventional (not segmented k-space) acquisitions, the acquisition time, T_2D_ is determined by the TR, the number of phase encoding steps, N_P_ and the number of signals averaged, NSA. For 3D imaging (**b**), a volume (or thick slice) is excited and then encoded in three dimensions by phase encoding and frequency encoding as for 2D imaging and additionally by phase encoding in the volume selection direction. Compared to 2D imaging, the acquisition time for 3D imaging, T_3D_, is increased by a factor equal to the number of phase encoding steps in the volume selection direction, N_S_. In practice, N_S_ is greater than expected as more slices are acquired than specified by the user and discarded after reconstruction. This because the tissue excitation at the edge of the volume is less well defined and these images may also suffer from image wrap which is analogous to the image wrap experienced in the 2D phase encoding direction.

The standard pulse sequence used for CE-MRA is the spoiled-gradient echo pulse sequence as described in part I [1] with the vendor-specific names FLASH (Siemens), T1-FFE (Philips) or SPGR (GE). A gradient echo pulse sequence is chosen because it is possible to achieve very short TR values for fast acquisition times and it exhibits bright-blood contrast in comparison with spin echo based pulse sequences. Spoiling is used to remove the influence of steady-state signal from the transverse component of magnetisation and to ensure T1-weighting. Both the minimum TE and TR are limited by the MR system hardware performance. The TE must be as short as possible to minimise signal from the short T2 and magnetic susceptibility effects of the contrast medium during the first pass stage. The minimum TR is the main parameter which determines the scan time for a given scanning geometry. It is therefore important to make the TR as short as possible. CE-MRA is normally acquired using a three-dimensional (3D) acquisition (Figure 27), where a volume (or thick slice) of tissue is excited at for each repetition, and then encoded in three dimensions [27]. In comparison to a 2D acquisition, a 3D acquisition allows thinner slices to be reconstructed which are also contiguous. This provides significantly improved spatial resolution in the slice direction, thus improving the quality of the multi-planar reformatting (MPR) and Maximum Intensity Projections (MIPs). Even though 3D acquisitions require a substantial increase in the number of repetitions, the scan times are still relatively short, because a very short TR is used. The very short TR also results in increased saturation (signal reduction) of the background tissue signal, which significantly improves the quality of the maximum intensity projections. 

### Key pulse sequence parameters for CE-MRA

The key geometry parameters of a spoiled gradient echo pulse sequence are chosen to give the best possible spatial resolution and image quality while providing adequate coverage of the imaging volume and minimising the acquisition time for patient breath-holding. Table 4 attempts to illustrate how these conflicting aims affect the choice of each parameter. In the ideal case it is desirable to generate both high SNR and high spatial resolution with a fast scan time. In practice, an improvement in one of these must be traded against the other two. This can be better appreciated by understanding the factors that determine SNR.

**Table 4:** Influence of geometry parameters for CE-MRA acquisitions

**Geometry Parameter**	**How is it chosen?**	**Why?**	**What is the limitation/ disadvantage?**
Acquisition Matrix, N_R_, in the readout direction (Base Resolution)	256 Minimum Increase to 512 preferred	To get best resolution in readout direction	512 matrix increases TE and therefore TR. (increases scan time)
Acquisition Matrix, N_P_, in the phase encoding direction	Maximise	To get best resolution in phase encoding direction	Increases Scan time (directly proportional)
	(depends on breath-hold period)		
			SNR decreases as square root of increase
No of slices, N_S_	Maximise	Increases coverage of volume thickness	Scan time (increases proportionally)
	(depends on breath-hold period, slice thickness and FOV_S_		
		Increases SNR as square root	
		(fixed slice thickness)	
Slice thickness, THK	Minimise for best resolution in the slice direction	Increases through-plane resolution	Decreases coverage
FOV_S_ = THKxN_S_			Decreases SNR (directly proportional)
Field of View (FOV_R_)	Optimise for desired in-plane coverage	Aim to get best in-plane resolution without getting too much foldover	If too small, foldover becomes a problem.
			SNR decreases with FOV_R_ (proportional to square).
Rectangular Field of View factor (RFOV)	Minimise for desired coverage in phase encoding direction	Reduces scan time	Foldover is increased
FOV_P_ = RFOVxFOV_R_			
		(N_P_ reduces in same proportion as RFOV)	SNR is decreased
			(Proportional to square root)
Zero filling/ interpolation	Apply in the slice direction	Doubles the number of reconstructed slices (but doesn’t improve resolution)	None

This table shows how different geometric parameters can be chosen to improve spatial resolution and coverage of the region of interest, together with the trade-off reflected in increased scan time or poorer signal to noise ration. Abbreviations are as follows: N_R_, N_P_, N_S_ = Acquisition matrix in the readout (frequency), phase and slice encoding directions respectively. FOV_R_, FOV_P_, FOV_S_ = Field of view in the readout (frequency), phase and slice encoding directions respectively. RFOV = rectangular field of view factor. THK = slice thickness.

In general SNR is proportional to the voxel volume and the square root of the number of phase encoding steps, while the spatial resolution is directly related to the voxel dimensions. For a 3D acquisition, the voxel dimension in the phase encoding direction is equal to the field of view in that direction divided by the number of pixels N_P_ in that direction, while the voxel dimension in the slice encoding dimension (the slice thickness) is equal to the field of view in that direction divided by the number of pixels (or slices) in that direction, N_S_.

The acquisition time is calculated from the TR the number of phase encoding steps in each direction, corresponding to the number of pixels and slices thus:

$$\text{Scan time}=\text{TR}.N_{P}.N_{S}$$

For a given field of view, increasing N_P_ and N_S_ gives a proportionate improvement in spatial resolution in each direction, but also increases the scan time in proportion to the product of the two. In each case the SNR is also decreased (proportional to the square root of N_P_ and N_S_). This is because the increase in SNR caused by the increased signal sampling (proportional to the square root of the product of N_P_ and N_S_) is more than offset by the decrease in SNR caused by the reduction in voxel volume (directly proportional to the product of N_P_ and N_S_).

Given the above constraints the best way to minimise the acquisition time whilst achieving high spatial resolution is to make the TR, and consequently the TE, as short as the MR system hardware and software will allow. The use of low flip angles, described in Part I [1], together with the reduction of T1 relaxation times by the use of contrast agent makes extremely short TR values of less than 5 ms possible.

The flip angle is chosen to maximise the signal for a particular TR and T1 value (known as the Ernst angle). In practice flip angle values in the range 30°-40° which are slightly higher than the Ernst angle are used to improve the saturation of the background tissues and hence maximise the contrast between them and the vessels.

### Timing of the start of acquisition after contrast injection

**Figure 28:**
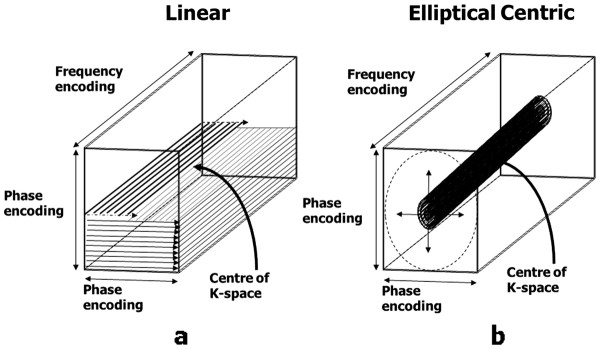
**k-space order for 3D image acquisitions.** The concept of k-space order for 2D image acquisitions was explained previously (see Part I, Figure 14) [1]. This diagram shows the two approaches to k-space ordering in 3D imaging that are equivalent to the linear and centric k-space orders in 2D imaging. In (**a**), the linear k-space order for 3D acquisitions begins and ends at the edge of k-space, passing through the centre of k-space at the mid-point of the acquisition. For each phase encoding step in one direction, (vertical in this example) the acquisition steps through all the phase encoding steps in the other direction (horizontal) so that the lines of k-space are filled layer by layer. In (**b**), the centric k-space order for 3D acquisitions begins at the centre of k-space and works outwards, adding layers in a pattern of concentric elliptical cylinders of increasing size. (The number of k-space lines in each phase encoding direction is usually different, hence the elliptical shape).

Achieving the correct timing of the start of the acquisition from the time of contrast injection is perhaps the most crucial aspect of CE-MRA as it strongly determines the quality of the result. The timing must take account not only of the acquisition time of the MRA pulse sequence, but also the order in which the lines of k-space are acquired during the 3D acquisition (the k-space order). The central lines of k-space primarily determine the image contrast [1,25]. The timing of the MRA acquisition must therefore be such that the central lines of k-space are acquired at the time of highest concentration of contrast agent within the vessels of interest. For 3D CE- MRA acquisitions, the two most common approaches to k-space order are linear, where the centre of k-space is acquired at the centre of data acquisition and elliptical centric [90,91], where the centre of k-space is acquired at the start of data acquisition (Figure 28). 

**Figure 29:**
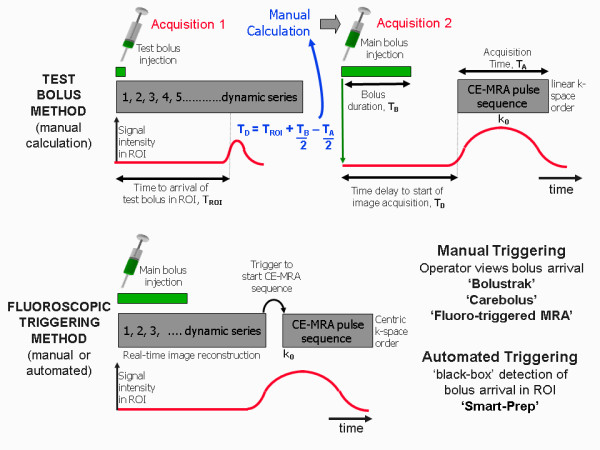
**The two principle methods used for timing of the start of the CE-MRA data acquisition.** For the test bolus method (top) a small bolus (1-2 ml) of contrast agent is first injected and a single-slice acquisition is used to acquire a dynamic series of images at approximately 1 second intervals in the region of interest (ROI). The image time series is analysed to determine the time at which the contrast bolus arrived in the ROI, T_ROI_. The time delay T_D_ for the CE-MRA acquisition is then manually calculated taking into account the time duration of the bolus, T_B_, the acquisition time of the MRA pulse sequence, T_A_ and the k-space order (in this case linear). For the fluoroscopic triggering method (bottom) the first acquisition acquires a dynamic series of images at approximately 1 sec intervals which are reconstructed in real time to allow detection of contrast arrival. On detection of bolus arrival, a trigger signal is generated either manually or automatically that causes the acquisition to switch to the CE-MRA pulse sequence. Manual triggering requires the operator to view the dynamic series of images in real time and to initiate the trigger once they judge that the contrast bolus has adequately progressed into the region of interest. Automated triggering monitors the signal intensity within a region of interest predefined by the operator on the dynamic image data set. The trigger signal is generated once the signal intensity rises above a threshold value.

There are two main approaches for timing the start of data acquisition (Figure 29), based either on a predetermined time delay measured using a small test bolus [92], or on the real-time monitoring of the contrast agent entering the region of interest to be imaged using a dynamic acquisition sometimes referred to as MR fluoroscopy [91,93], with vendor specific names BOLUSTRAK (Philips), CareBolus (Siemens), Fluoro Triggered MRA (GE). The test bolus method involves the injection of a small volume of contrast agent (typically 2-3 ml), followed immediately by a larger volume of saline flush. Single-slice dynamic scans over the artery of interest are acquired typically using a spoiled 2D gradient echo pulse sequence with saturation pulses selectively applied both above and below the slice to eliminate “inflow” effects. A single-shot IR gradient echo sequence is also commonly used, with TI set to null the signal from blood. The delay time from the start of injection to the start of scan can then be calculated by using the ‘contrast arrival time’ for the test bolus, recorded by viewing the dynamic scans. The aim is to match the centre of the bolus, giving maximum concentration, to coincide with the centre of k-space acquisition. This calculation must therefore also take account of the bolus injection duration, the acquisition duration and the k-space order, which is normally selected as linear for this approach. An advantage of the test-bolus approach is that it allows the operator to verify that the injection system is working and the IV access is patent prior to proceeding to the high resolution acquisition. It also provides the operator with advance knowledge of the individual patient’s circulation time so that the main acquisition parameters can be optimised. These are particularly useful features for operators who have limited experience with CE-MRA. 

The MR fluoroscopy technique may use either a manual or automated trigger to start the CE-MRA acquisition. The manual trigger method is based upon visual bolus detection [94]. In this case, a single thick slice is positioned to include the vessels of interest and images are acquired and updated continually with a one second temporal resolution. There are a number of potential advantages of this approach. First, the operator watches for the arrival of the contrast agent and triggers the 3D CE-MRA acquisition manually at the appropriate time. The operator therefore gains an impression of how fast the circulation is. This is useful in patients with extremely slow flow (typically patients with abdominal aortic aneurysms) in whom triggering of the 3D acquisition as soon as contrast arrives within the upper abdominal aorta would result in artefacts due to delayed filling of the iliac arteries. As a single thick slice is used for bolus detection, the slice encompasses a large amount of the anatomy. Therefore, sequential enhancement of the right side of the heart, pulmonary circulation, left heart chambers and aorta is easily visualized. This sequential enhancement is useful as the operator has more warning to co-ordinate the breathing instructions accordingly. The scanner briefly falls silent after aborting the 2D fluoroscopic scan. Communication with the patient for the instruction to breath-hold is therefore unhindered. Real-time subtraction of the 2D fluoroscopic images eliminates the background tissue signal and improves visualization of contrast arrival.

For the automated triggering approach [95] the MR system continuously monitors the signal intensity within a single large voxel placed over an artery (e.g. upper abdominal aorta) within the region-of-interest (ROI). The scan is triggered when the signal intensity rises above a predetermined threshold value, indicating the arrival of contrast medium in the selected artery. This approach removes user dependence from the process but there is no possibility for the operator to interactively select a slightly longer scan delay time, for example in a patient with extremely slow flow in whom there may be a prolonged delay between arrival of contrast within the upper abdominal aorta and the iliac arteries.

### Time-resolved contrast-enhanced MRA techniques

**Figure 30:**
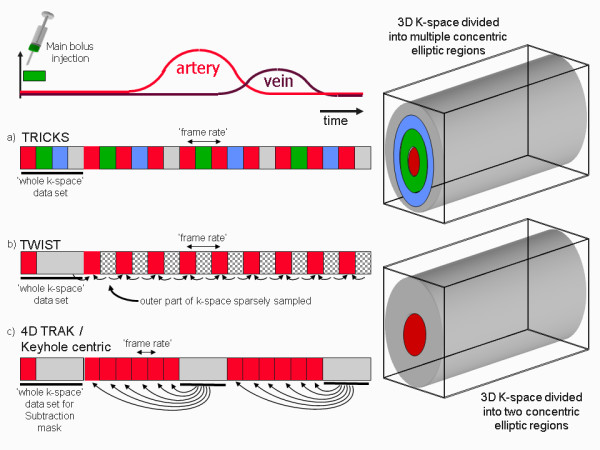
**This diagram shows the time- resolved MRA approaches offered by three vendors.** In each case, the effective frame rate is defined as the time interval between central zone acquisitions as this determines how often the image contrast is updated. In (**a**) the vendor implementation of time resolved imaging with contrast kinetics (TRICKS), divides 3D k-space into four elliptical concentric zones. Data is acquired initially for all four zones and subsequently the acquisition of the central zone is interleaved with each of the three outer zones in sequence. The solutions from two other vendors divide k-space into just two zones. In (**b**) the TWIST technique initially samples the whole of k-space and subsequently acquires the central zone interleaved with the outer zone. As the outer zone is much larger, although k-space lines are updated over the full extent of the outer zone, many lines are skipped. The missing lines are then updated in subsequent outer-zone acquisitions. In (**c**) the 4D-TRAK technique acquires the whole of k-space at the beginning, followed by between 4–6 consecutive acquisitions of the central zone, after which the outer zone of k-space is sampled once more. The initial whole k-space acquisition is used as a mask which is subtracted from later reconstructions to remove the background tissue signal. Subsequent outer zone acquisitions are combined with the preceding 6 central zone acquisitions (indicated by the curved arrows) to reconstruct time resolved image data sets.

The CE-MRA techniques described so far result in a single 3D image data set at one time point during the passage of contrast through the region of interest. Limited dynamic information can be obtained by simply repeating the same acquisition but the maximum ‘frame rate’ for such an approach is limited by the relatively long acquisition time (typically 10–15 seconds). Time resolved CE-MRA techniques were developed to overcome this limitation, reducing the time interval between successive 3D MRA data sets (the temporal resolution) to just a few seconds whilst maintaining high spatial resolution. The technique was first published as time resolved imaging of contrast kinetics (TRICKS) and introduced the idea of dividing the 3D k-space into several zones [96]. The image data within each k-space zone is acquired at different selected time periods, however the zone at the central part of k-space is re-acquired more frequently than the other zones, updating the contrast information more rapidly than the other higher spatial frequency information (Figure 30). Each time the contrast information is updated, the whole k-space data is used to reconstruct a new image dataset. Vendor specific time-resolved MRA techniques are now available with GE adopting the TRICKS method. The keyhole-centric and 4D-Trak (Philips) and TWIST techniques (Siemens) divide k-space into just two zones and use differing approaches to fill the outer part of k-space. Time resolved CE-MRA techniques are of particular advantage when assessing congenital vascular anomalies and arterio-venous malformations. 

### Non-Contrast Enhanced MRA techniques

Non-contrast-enhanced MRA (NCE-MRA) techniques rely on changes in the MR signal that are caused by the motion of blood through or within the image plane. There are three principal flow effects that can occur, depending on the pulse sequence that is being used: The spin washout effect described in Part 1 of this review [1] gives a ‘dark’ or ‘black’ blood appearance and is characteristic of spin echo pulse sequences. This effect has sometimes been used as the basis for ‘black blood‘ angiography. The second effect, flow-related enhancement, gives rise to the bright blood contrast observed with spoiled gradient echo pulse sequences and is also described in Part 1 [1]. This is the principal effect that is used as the basis for time-of-flight (TOF) MRA [97] which for body applications has been largely replaced by CE-MRA, but still has some application in the head for imaging of the circle of Willis and the sagittal sinus [97,98].

The third effect, phase-related signal loss gives rise to the appearance of signal voids in cine gradient echo pulse sequences in the presence of flow jets and turbulence. This is due to the intrinsic flow sensitivity of pulse sequences and is described in more detail in the flow velocity mapping section (see later). The same principle that causes this signal loss is used as the basis for phase contrast angiography or PCA [99,100]. In this case, the gradient pulses are designed to produce phase changes which are typically less than 180° for a given velocity range. In this way, the signal is not completely de-phased, and the phase information is preserved during the image reconstruction. Since the phase of the signal can depend on many factors, it is necessary to perform at least two acquisitions so that the phase changes due to the other factors can be removed by subtracting one image acquisition from the other. In practice, an image data set is first acquired using a pulse sequence that is relatively insensitive to flow (or flow compensated), followed by one that is sensitive to flow over a particular range of velocities in one chosen direction. To image blood flow in all three directions the flow sensitive acquisition is repeated in each of the other two directions. The signal data from the flow compensated acquisition is then subtracted from each of the flow sensitive acquisitions, giving a phase contrast image for each flow direction. Since the phase is unchanged for static protons, the subtraction completely suppresses the signal from the background tissue. The final step in the image reconstruction is to combine the phase contrast data sets for each direction to calculate a ‘speed’ image. The signal intensity on PCA images is related to velocity. The operator prior to the scan sets the flow sensitivity of the pulse sequence by defining the maximum velocity range (VENC; +/− max velocity).

More recently, the same principle used for PCA has been modified to provide a NCE-MRA technique to image the peripheral arteries, exploiting the difference in flow velocities between systole and diastole. Whereas PC-MRA is normally used in combination with a 2D or 3D gradient echo pulse sequence, this method uses two 3D fast (or turbo) spin echo acquisitions, synchronised first to systole and then diastole [98,99,101]. Phase-related signal loss relating to higher velocities in systole is not present in diastole and the difference in signal is used to generate an arteriogram by subtraction of the two data sets. Techniques that apply this principle are now available commercially, implemented as Flow-Spoiled Fresh Blood Imaging (FBI) by Toshiba, Native SPACE MRA by Siemens, TRANCE by Philips and InHance 2D Inflow by GE.

A further recent development is the use of both 2D and 3D bSSFP pulse sequences to perform NCE-MRA [19,99]. The combination of their intrinsically high signal from blood and the use of fat suppression techniques provides a simple, flow-independent method of imaging vessels. Vendor implementations of this approach include FIESTA with Fat Sat, Balanced FFE with SPIR, True FISP with Fat Sat. The main disadvantage of this approach is that both the arterial and venous system is imaged, as well as other fluid-filled ducts, although the venous suppression techniques used in TOF-MRA can also be applied here to remove unwanted venous signal. Balanced SSFP techniques have also been combined with a technique known as arterial spin labelling (ASL) to provide high quality arteriograms of both the renal and carotid arteries [19,98,99]. Vendor implementations of this technique include time-SLIP by Toshiba, Native TrueFISP by Siemens, b-TRANCE by Philips and InHance Inflow IR by GE.

### Coronary MRA with respiratory gating using navigator echoes

**Figure 31:**
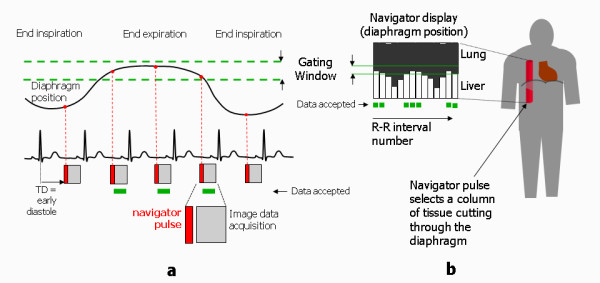
**Respiratory gating and navigator echoes.** This figure illustrates the principle behind the use of navigator echoes to gate the image data acquisition according to a particular time period within the respiratory cycle. The diagram in (**a**) shows a cardiac triggered data acquisition with a trigger delay chosen at mid-diastole to minimise the effect of cardiac motion. A curve representing the diaphragm position during respiratory motion is shown above. The effect of respiratory motion is limited by gating the data acquisition, so that image data is only accepted when the diaphragm position lies within a predefined ‘window’ corresponding to end expiration. A navigator pulse (shown in red) is applied immediately before the image data acquisition to excite a column of tissue cutting through the right hemi-diaphragm at right angles (**b**). The resultant navigator echo is frequency encoded along the length of the column and the navigator echo signal is analysed using a Fourier transform to produce a line of signal. The line signal from each successive R-R interval is added to a navigator display. A computer algorithm detects where the signal intensity changes from a high value (liver) to a low value (lung), representative of the diaphragm position. Where the diaphragm position falls within a predefined gating window the image data acquisition is accepted (indicated by the green dashes). Where the diaphragm position falls outside the gating window the data acquired is rejected and the acquisition is repeated until the diaphragm position again falls within the gating window.

**Figure 32:**
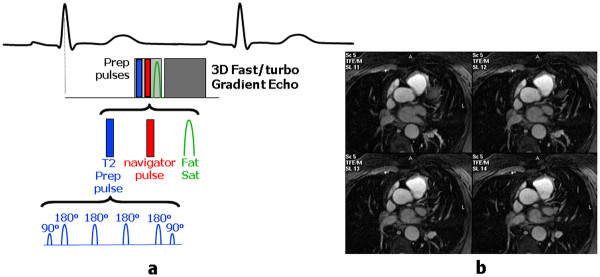
**Respiratory navigator-gated 3D Coronary MRA pulse sequence.** The key features of a navigator-gated 3D coronary MRA pulse sequence are shown in (**a**). The image data acquisition is typically performed using a 3D fast or turbo gradient echo (FGE) pulse sequence with a trigger delay set to acquire during mid-diastole. The image data acquisition is typically preceded by three preparation pulses, a T2-preparation pulse, the navigator pulse (see Figure 31) and a frequency-selective fat suppression pulse (see Figure 2 and Figure 5). The fat suppression pulse is necessary to suppress the signal from fat surrounding the coronary arteries and is applied immediately before the image data acquisition to maximise the effectiveness of fat suppression. The T2 preparation pulse is used to reduce the signal from the myocardial muscle (short T2) relative to that of the blood (long T2). This prep pulse consists of a 90° rf pulse followed by a series of 180° rf pulses, similar to a multi-echo spin echo pulse sequence. This produces magnetisation in the transverse plane that is T2-weighted. The T2-weighted transverse magnetisation is then rotated back to the z-axis by a second 90° rf pulse, resulting in z-magnetisation for myocardium that is reduced relative to that of the blood within the coronary arteries. This improves the contrast of the resultant MRA images. Four slices from a 3D coronary MRA dataset are shown in (b). Note the absence of fat signal from around the coronary arteries and the reduced signal contribution from the myocardium.

Imaging of the coronary arteries is one of the most challenging applications of cardiac MR imaging due to their small size and their motion with the cardiac and respiratory cycles. The most common pulse sequence used to image the coronary vessel lumen is a three dimensional (3D) gradient echo pulse sequence, combined with a series of preparation pulses to optimise the vessel contrast [20,102]. The use of a gradient echo pulse sequence for data acquisition provides a relatively enhanced signal from flowing blood. The 3D data acquisition allows thin slices to be acquired to achieve a high resolution data set. The acquisition of a 3D data set with high resolution in all three directions requires a long acquisition time, even when using a fast or turbo gradient echo sequence. This acquisition method cannot therefore be combined with breath-holding and the acquisition must instead be both cardiac triggered and respiratory gated. Respiratory gating requires the patient’s respiratory cycle to be monitored. The data acquisition is then controlled or gated (Figure 31a), such that data is acquired predominantly from one part of the respiratory cycle. This is usually chosen as end expiration as it is assumed that the respiratory cycle for the majority of patients dwells there for longer periods. The simplest way to monitor the respiratory cycle is to use a pneumatic bellows system held against the abdomen with an adjustable belt. For the high spatial resolution required to image the coronary arteries, however, a more accurate method is employed for real-time monitoring of the diaphragm position using navigator echoes (Figure 31b). Navigator echoes are generated using a specially designed rf pulse (or pulses) to excite a column of tissue through the diaphragm immediately before each image data acquisition. A line of signal is reconstructed from each navigator echo and displayed as a trace. The boundary between the low signal intensity in the lung and the relatively high signal intensity in the liver creates an edge that can easily be detected and used as a gating signal which accurately reflects the diaphragm position and is used to determine whether the data is accepted or rejected. The accuracy of this method allows narrow gating windows to be set (typically 5 mm) for high resolution applications such as coronary artery imaging. Typically the position of the gating window is set automatically based upon an in-line analysis of the respiratory navigator prior to the start of each image data acquisition. The magnetisation preparation scheme for coronary imaging includes the rf navigator pulse and a frequency selective fat suppression pulse that is used to suppress the fat signal surrounding the coronary arteries (Figure 32a). The image data acquisition is also preceded by a T2-preparation scheme [102]. This scheme uses the same series of pulses as a fast spin echo train (90°-180°-180°-180°-180°) which produces a transverse magnetisation that is T2-weighted. The final pulse in this T2-preparation scheme is a 90° that restores this magnetisation along the z-axis. This component of the preparation scheme helps to suppress the signal from the myocardium (short T2) relative to the blood signal (long T2). The 3D fast or turbo gradient echo pulse sequence is chosen for data acquisition to enable multiple contiguous thin slices to be acquired, allowing curved multi-planar reconstruction of images along the path of each coronary artery (Figure 32b). Coronary MRA may be performed either using two targeted thin volumes to separately cover the left and right coronary artery systems [20], or to acquire a much larger single 3D volume acquisition of the whole heart, from which reconstructed views of each artery are obtained [103]. 

### Flow velocity mapping

#### Intrinsic flow sensitivity of pulse sequences

**Figure 33:**
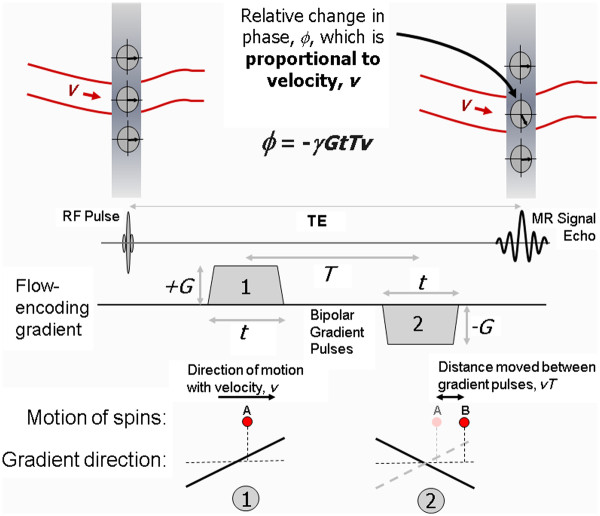
**This diagram illustrates how velocity-related phase shifts are caused by blood flowing along the direction of two equal but opposite magnetic field gradients applied in succession, (bipolar gradient pulses).** Two gradient pulses are shown with gradient amplitudes *G* and duration *t,* separated by a time *T*. Immediately after the rf pulse is applied, both moving and stationary spins have the same phase (top left). When the first positive gradient pulse is applied (1), spins at position A experience an increase in magnetic field due to their position along the gradient. When the second negative gradient pulse is applied [2], stationary spins that remain at position A experience an equal decrease in magnetic field, causing the spins to move back into phase with one another. Spins in moving blood with velocity *v*, however, will have moved to position B, and experience an additional decrease in magnetic field that is proportional to the distance moved, *vT,* from position A. The signal from moving blood therefore acquires a phase shift, ϕ, relative to that of stationary tissue, that is proportional to velocity, *v* as shown (top right). Bipolar gradient pulses are used to design velocity-sensitive pulse sequences. From the equation shown (top centre) it can be seen that the velocity-related phase shift, ϕ, is also proportional to amplitude G, the duration, t, and the separation, T, of the gradient pulses. Velocity sensitive pulse sequences are designed to be sensitive over different velocity ranges through careful choice of these gradient pulse parameters.

Qualitative assessment of blood flow patterns can be performed using cine gradient echo pulse sequences. Spoiled gradient echo pulse sequences (in comparison to bSSPF sequences) are particularly useful for the visualisation of flow jets associated with regurgitant and stenotic valves, stenotic vessels and septal defects, due to their inherent sensitivity to the presence of these flow jets [104,105]. This sensitivity arises due to the motion of spins within the flowing blood along the magnetic field gradients that are applied as part of the gradient echo pulse sequence [23,106]. The formation of the gradient echo requires any de-phasing of the magnetisation caused by either the slice selection or frequency encoding gradients to be reversed at the echo time (TE). This is achieved by applying two gradients along the same direction but with opposite signs [1]. This combination is known as a bipolar gradient pulse pair. The second gradient reverses (re-phases) the de-phasing caused by the first gradient for stationary tissue, but for spins within flowing blood that change their position along the gradient during the interval between the two gradients (Figure 33), the complete reversal by the second gradient of the phase changes caused by the first gradient is not achieved. As a result, the phase of the transverse magnetisation within flowing blood after the two gradient pulses is different from the phase of transverse magnetisation within stationary tissue and the difference is proportional to the velocity of the blood in the direction of the applied gradient [22]. For a certain velocity, the size of this phase difference depends on the flow sensitivity of the pulse sequence. This depends on the amplitude (or slope) of the bipolar gradients G, their duration, t and the time interval between the two gradient pulses, T (Figure 33). 

### Appearance of flow voids

**Figure 34:**
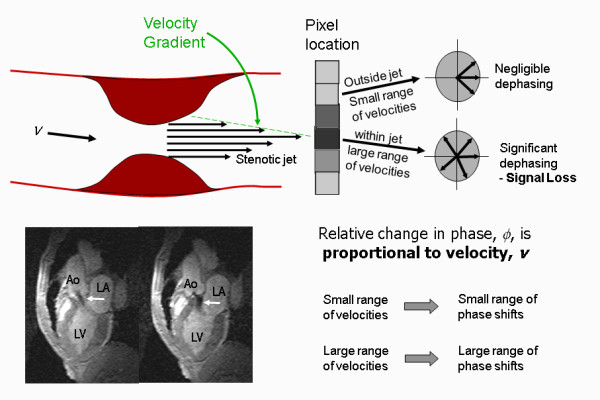
**Signal losses caused by velocity gradients.** This diagram illustrates how signal losses occur in the presence of stenotic or regurgitant flow jets. The two images (bottom left) show signal loss (white arrows) caused by aortic valve regurgitation at two end systole and early diastole. Flow jets consist of a large range of velocities, from very high velocities at the centre of the jet, to relatively low velocities at the edge of the jet (top left). The change in velocity from the centre to the edge of the jet is often referred to as a velocity gradient. Figure 33 shows that when bipolar gradients are applied within imaging pulse sequences, velocity related phase shifts occur. Where there is a large range of velocities due to a velocity gradient within a pixel, this results in a large range of phase shifts (right), causing significant dephasing and therefore signal loss within that pixel. Outside the jet there is only a small range of velocities leading to negligible dephasing and no signal loss.

Gradient Echo-based pulse sequences normally produce images with a ‘bright-blood’ appearance. In the presence of flow jets however, a signal void is often seen at the location of the jet (Figure 34). This effect is commonly observed when imaging regurgitant valves, stenotic vessels or flow through septal defects [105]. The signal void is caused by a de-phasing of the magnetisation in the presence of the jet. This is a consequence of the velocity-related phase shift caused by motion along the magnetic field gradients as described in the previous section. The flow jet contains a large range of velocities (sometimes referred to as a velocity gradient). This causes a large range of phase shifts, causing de-phasing of the transverse magnetisation and therefore resulting in signal loss (Figure 34). The flow-related signal void is often qualitatively related to the size and severity of the flow jet and is sometimes used to grade the severity of regurgitation [107]. Qualitative assessment of this kind must be done with caution, as the size of the signal void also depends on the pulse sequence type, the echo time and a number of other parameters that affect the imaging gradients strength and duration. For example, increasing the echo time increases the apparent size of the flow jet [108]. Additionally, the same flow jet visualised using a bSSFP pulse sequence is smaller than when visualised using a spoiled gradient echo pulse sequence with equivalent imaging parameters. Common solutions to remove or reduce flow-related signal loss are to reduce the echo time, use flow compensation or use a bSSFP pulse sequence that is less flow sensitive than a spoiled gradient echo pulse sequence. It has been shown that for spoiled gradient echo pulse sequences, a TE of less than 3.6 ms significantly reduces signal loss [105,109] but may require use of a high receiver bandwidth, and / or partial (asymmetric) echo sampling, both of which reduce SNR. Flow compensation, (also known as Gradient Moment Nulling or Gradient Motion Rephasing), can be applied to reduce the flow sensitivity of a pulse sequence. It is normally achieved by adding one or more gradient pulses to the pulse sequence where there is already a bipolar gradient pulse. The purpose of the additional gradient pulse(s) is to reduce or nullify the velocity-dependent phase shift caused by motion along the gradient. This reduces flow de-phasing and ghosting artefacts from pulsatile flow, but increases the echo time which may negate any benefit in the presence of signal loss caused by flow jets. 

### Flow velocity encoding and velocity mapping

This inherent flow sensitivity is exploited to enable the quantification of blood flow velocity by generating images, known as phase maps, in which pixel intensity depends upon the phase of the transverse magnetisation, rather than its magnitude [23,24]. There are however a number of potential causes of relative change in phase of the transverse magnetisation. These include phase changes due to motion along more than one gradient direction (arising from velocity components in these other directions) and phase changes due to magnetic field inhomogeneities.

The phase changes due to the above causes must be accounted for in order to isolate the change that is due to motion along the desired gradient direction. This is achieved by performing two consecutive acquisitions for each phase encoding step. The two acquisitions are identical other than that they have different flow sensitivities in the chosen direction of flow measurement, known as the velocity encoding direction. The flow sensitivity is determined by the amplitude, duration and time separation of the bipolar flow-encoding gradients in that direction. Once the image data acquisition is complete, phase maps from the two acquisitions are calculated and subtracted to produce a velocity map. The subtracted velocity map contains only phase shifts that are related to velocity components in the flow-encoding direction. Phase changes due to other causes, including velocity components in other directions and magnetic field inhomogeneities are removed by the subtraction.

**Figure 35:**
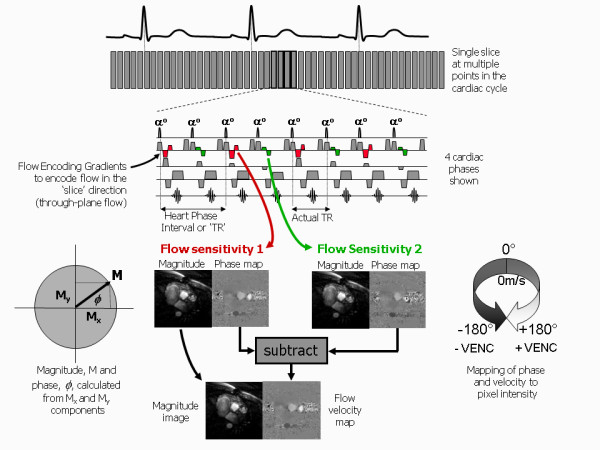
**A retrospectively-gated, cine spoiled gradient echo pulse sequence (top) is shown in detail for four cardiac phases.** In this example the standard imaging gradients are modified in the slice-selection direction to encode flow velocity components perpendicular to the image plane. For each cardiac phase the gradient echo pulse sequence is acquired twice, with two different flow sensitivities, indicated here by the red and green gradient pulses. The heart phase interval or ‘effective TR’ of the velocity encoded sequence is double the actual TR of the gradient echo pulse sequence. The M_x_ and M_y_ components of the MR signal are used to calculate both the signal magnitude, M, and the signal phase, ϕ, for each pixel. The phase maps for the two different flow sensitivities are subtracted to remove background phase shifts, creating a velocity map, which contains only velocity-related phase shifts due to the difference in flow velocity sensitivity between the two acquisitions. The flow sensitivities are chosen such that a subtracted phase difference of 180° corresponds to a predefined maximum velocity or VENC. The sign of the subtracted phase indicates the direction of flow along the encoded direction. The value of relative phase shift is mapped onto the pixel intensity scale such that a zero subtracted phase (stationary tissue) maps on to the centre of the pixel intensity scale (mid-grey). Positive and negative subtracted phases (corresponding to opposite directions of flow) are mapped onto higher and lower pixel intensities, respectively.

Velocity maps are generally displayed using a grey scale with stationary tissue being displayed as mid-grey, with velocities in forward (positive) and reverse (negative) directions being represented as higher (towards white) and lower (towards black) pixel intensities (Figure 35).

**Figure 36:**
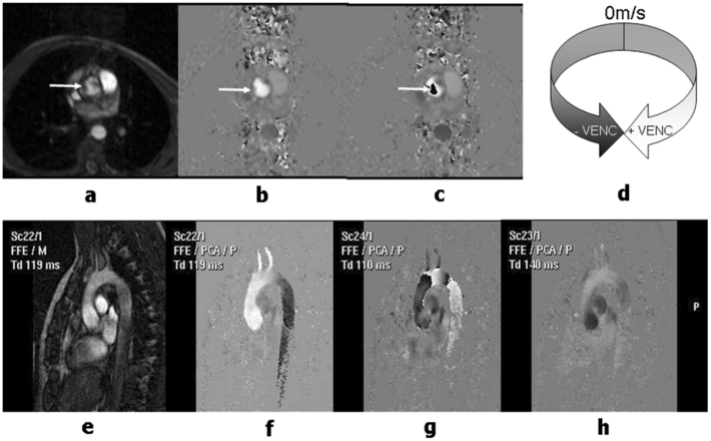
**This figure illustrates some of the common pitfalls encountered with velocity-encoded MR imaging.** Images (**a**) and (**b**) show a transaxial magnitude image and velocity map with ‘through-plane’ velocity encoding demonstrating forward flow in the ascending aorta (arrows). In (**b**), the maximum velocity is within the VENC chosen for the velocity-encoded acquisition, so that the phase shifts for all the pixels within the ascending aorta are less than 180° and are shown as high pixel intensities. In image (**c**), the VENC is set too low and the pixels at the centre of the ascending aorta (arrow) appear as negative velocities. This artefact, known as ‘velocity aliasing’ arises because phase shifts greater than 180° are interpreted as negative phase shifts and are mapped on to the lower pixel intensity scale (**d**). Images (**e**) and (**f**) show a magnitude image and velocity map acquired in an oblique sagittal plane to demonstrate flow in the aortic arch. The velocity encoding direction is chosen as ‘in-plane’ to demonstrate velocity components in the head-feet direction. In (**f**), the maximum velocity in both the ascending and descending aorta is lower than the VENC so that no aliasing is visible. For image (**g**) the VENC has been set too low resulting in velocity aliasing. For image (**h**) the velocity encoding direction has been incorrectly chosen to encode velocity components through the image plane, so that only relatively low transverse components of blood flow velocity are visible in the aorta.

The maximum measurable velocity range, often referred to as the VENC, is defined by the MR operator. It is determined by the difference in the flow sensitivities of the two acquisitions. Selecting a VENC that is too low is a common pitfall of velocity-encoded cine MR imaging [23,24]. The imaging of blood flow with velocities that are higher than the chosen VENC results in aliasing of the measured velocity value, with positive velocities being displayed as negative velocities and vice versa (Figure 36). The presence of flow jets or turbulence presents a further pitfall when attempting to quantify blood flow velocities as they can cause signal loss. At low signal magnitudes, calculation of the signal phase becomes unreliable, resulting in spurious velocity measurements [105,109]. This pitfall can be avoided by selecting a short echo time (below 3.6 ms) for the cine velocity mapping gradient echo pulse sequence. Another common pitfall arises when the wrong flow encoding direction is selected by the operator (Figure 36). More detailed considerations relating to acquisition and analysis of velocity-encoded cine MR image data is beyond the scope of this review but are covered elsewhere [23,24,105]. 

## Conclusion

This review has outlined the key physical principles that underlie the more advanced cardiac MR imaging techniques most commonly used in clinical practice. The basic principles of oedema imaging, myocardial cine tagging, myocardial perfusion, late enhancement imaging, magnetic resonance angiography and velocity mapping have been explained. Key imaging parameters have been defined, explaining their influence on image contrast, resolution and acquisition time. Where appropriate, common pitfalls have been discussed and the causes and remedies of common image artefacts have been explained. Further detailed reading is provided through the provision of key references. This review should be a useful resource from clinicians who wish to gain a greater understanding of the underlying physics of CMR.

## Competing interests

The authors declare that they have no competing interests.

## Authors' contributions

AR, JDB and JPR drafted, read, revised and approved the final manuscript.

## Supplementary Material

Additional file 1 **Perfusion defect movie.** Dynamic Contrast Enhanced (DCE-MRI) stress perfusion image movie. Contrast agent causes signal enhancement in the right ventricle followed by the left ventricle followed by the healthy myocardium. Coronary artery disease reduces or obstructs flow to the septal and anterior myocardium resulting in an area of hypo-enhancment (relative to the enhanced myocardium), known as a perfusion defect. Click here for file

Additional file 2 **Maximum intensity projection (MIP).** A PowerPoint animation showing how a projection angiogram is formed using a maximum intensity projection (MIP). In this simplified example a lateral projection of a simulated bifurcating vessel is generated from a series of transaxial slices. Each mouse click projects the maximum pixel data encountered within a single slice on to a single row of pixels. This process continues until all the slices in the MRA volume have been projected onto the final MIP image. Click here for file
