# Supplementary material for: Cardiovascular magnetic resonance physics for clinicians: part II
Source: J Cardiovasc Magn Reson. 2012 Sep 20;14(1):66. doi: 10.1186/1532-429X-14-66 (PMC3533879; doi:10.1186/1532-429X-14-66)
Supplement: Additional file 2 — Maximum intensity projection (MIP). A PowerPoint animation showing how a projection angiogram is formed using a maximum intensity projection (MIP). In this simplified example a lateral projection of a simulated bifurcating vessel is generated from a series of transaxial slices. Each mouse click projects the maximum pixel data encountered within a single slice on to a single row of pixels. This process continues until all the slices in the MRA volume have been projected onto the final MIP image. [file 1532-429X-14-66-S2.pptx]

## Slide 1
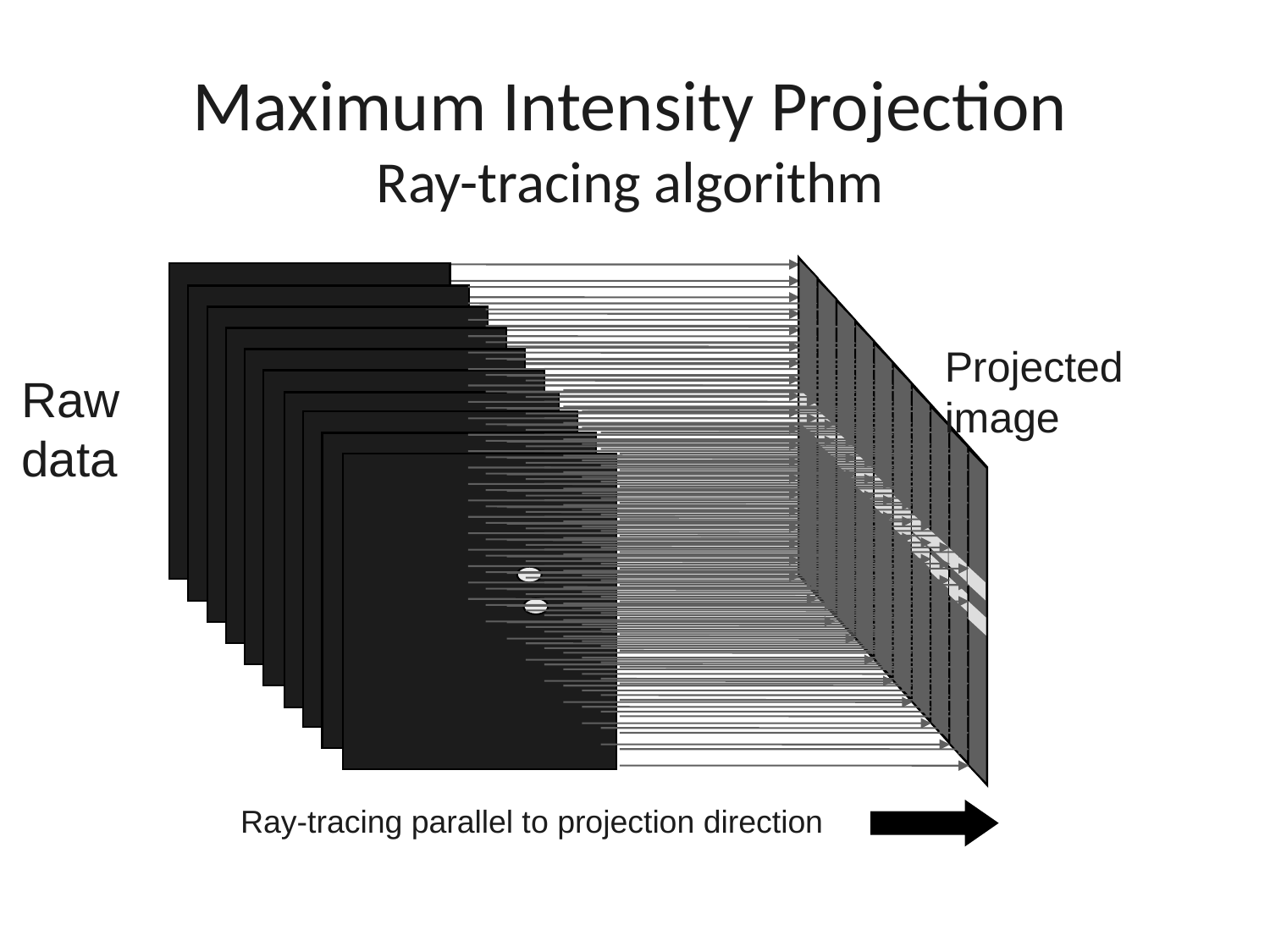

# Maximum Intensity Projection Ray-tracing algorithm
Projected image
Raw data
Ray-tracing parallel to projection direction
